# Supplementary figures and images for: Making the best of a bad sample: Comparison of DNA extraction and quantification methods using sub-optimally stored Ixodes ricinus ticks
Source: PLoS One. 2025 May 29;20(5):e0323251. doi: 10.1371/journal.pone.0323251 (PMC12121741; doi:10.1371/journal.pone.0323251)

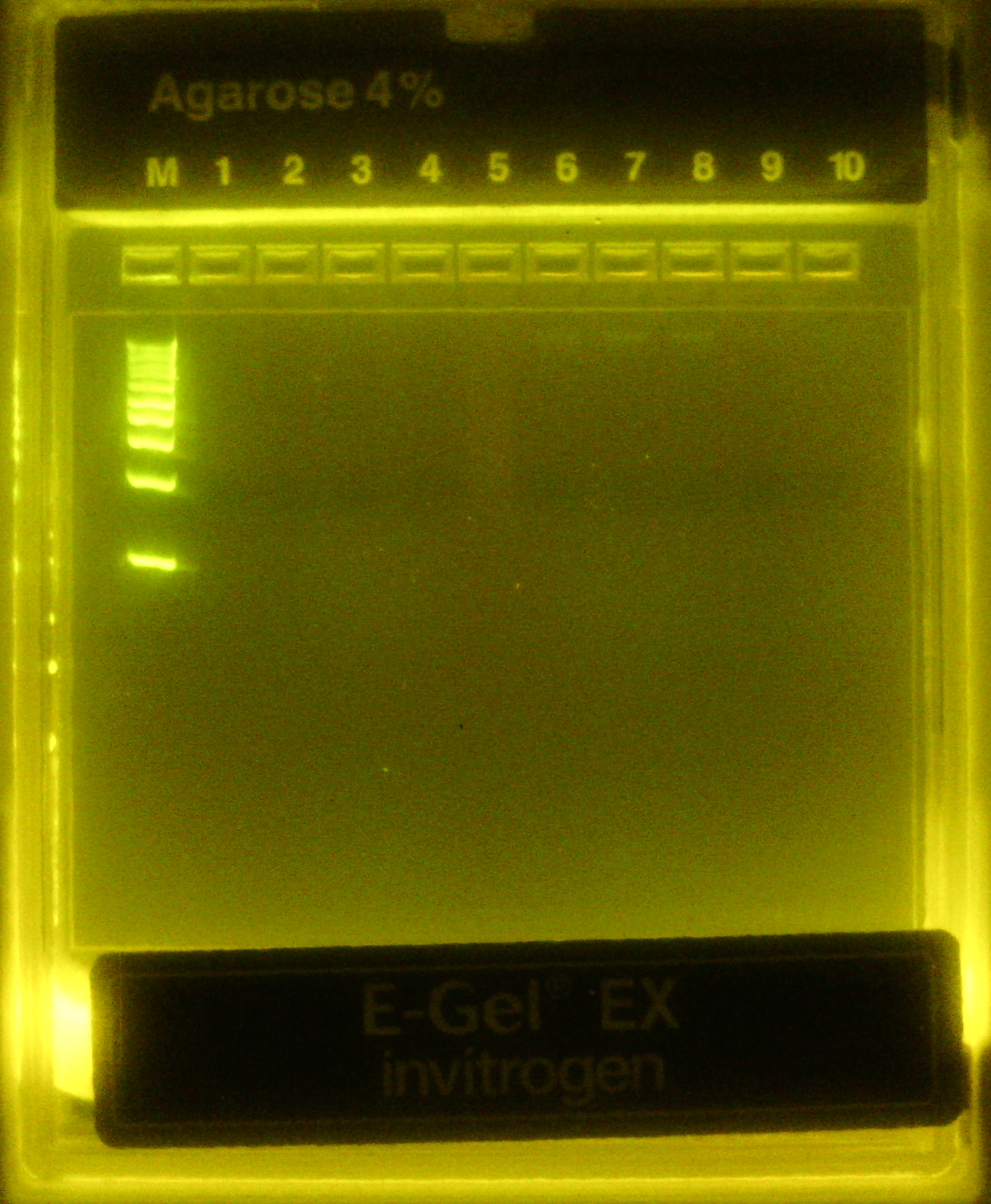

Supplement: S1 Fig — From well 1–10: AC-N, AC-N, AC-N, AC-N, AC-N, QMK-N, QMK-N, QMK-N, QMK-N, QBT-N. (JPG) [file pone.0323251.s001.JPG]

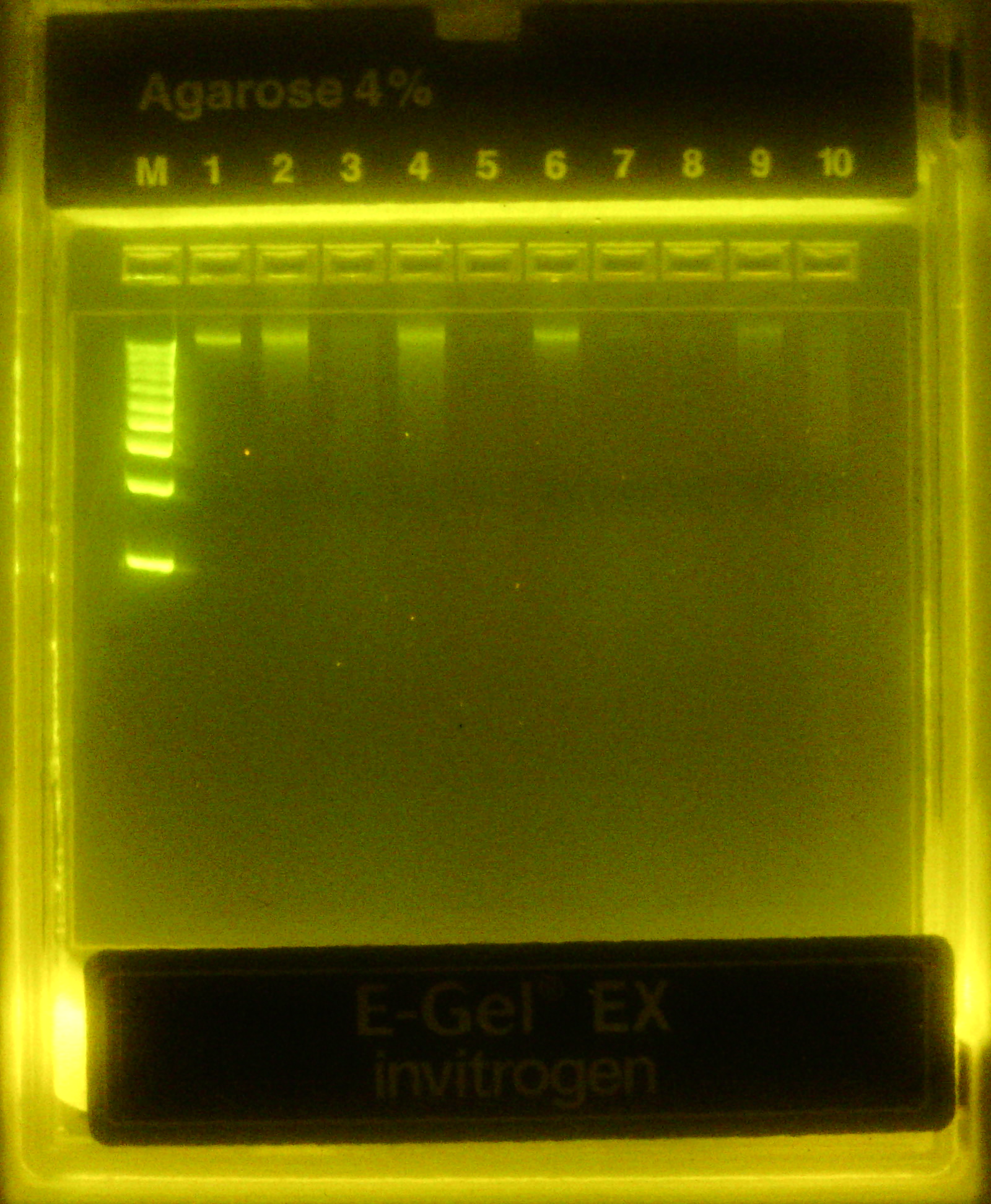

Supplement: S2 Fig — From well 1–10: QMK-N, QMK-N, QBT-N, QMK-N, QBT-N, QBT-N, QBT-N, QBT-N, QBT-N, ANC-N. (JPG) [file pone.0323251.s002.JPG]

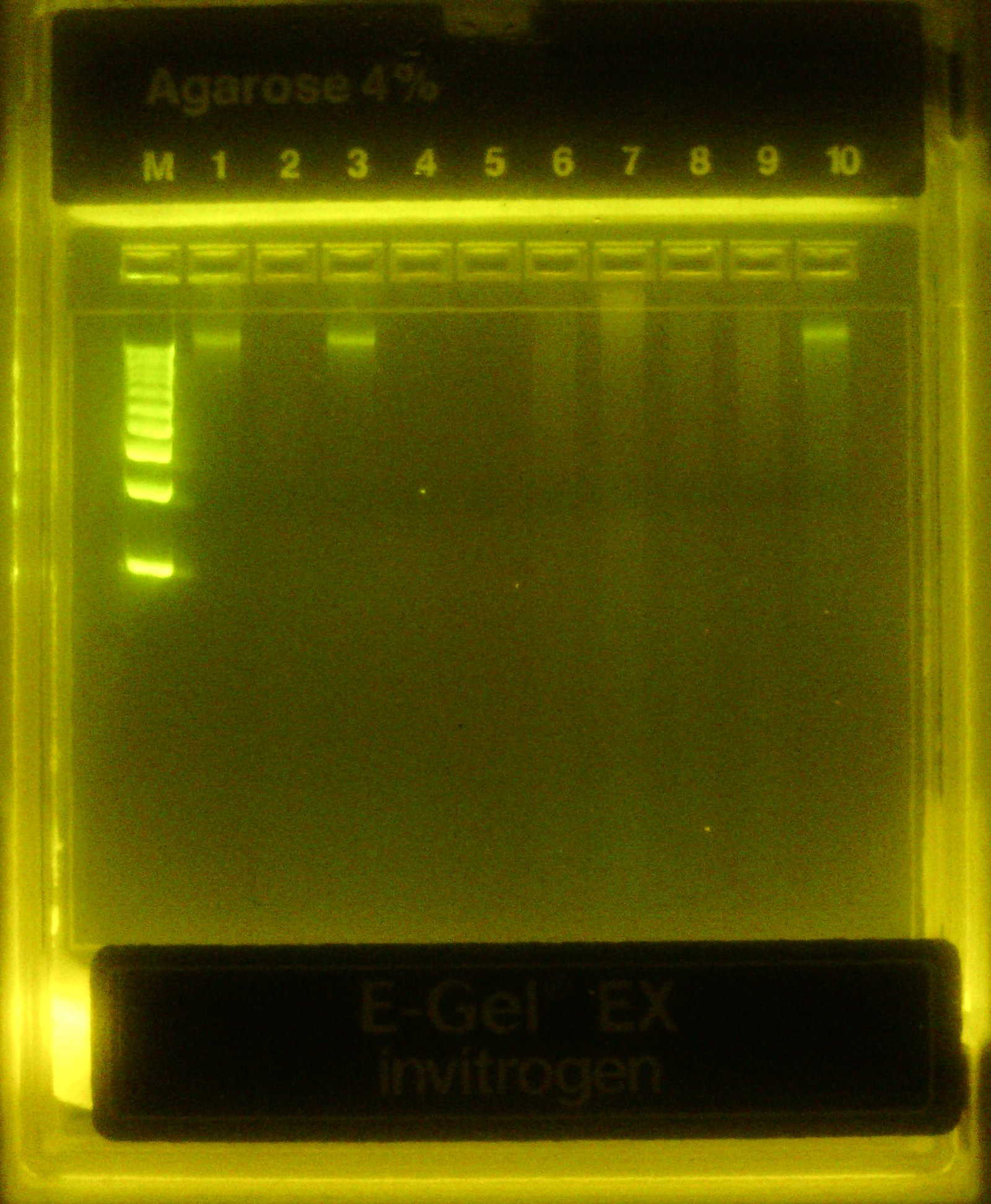

Supplement: S3 Fig — From well 1–10: QMK-N, ANC-N, QMK-N, QBT-N, QBT-N, ANC-N, AC-F, ANC-N, ANC-N, QMK-N. (JPG) [file pone.0323251.s003.JPG]

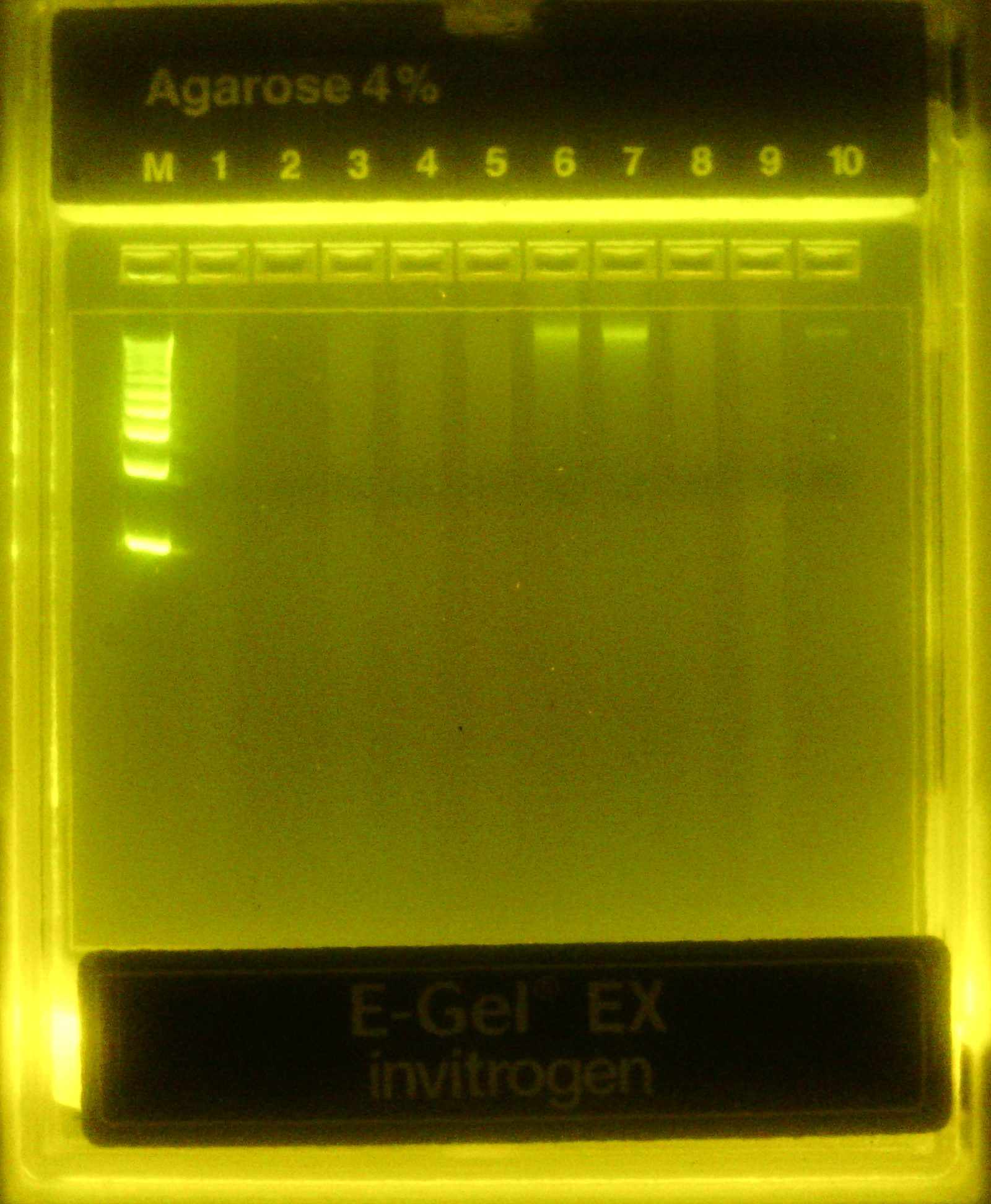

Supplement: S4 Fig — From well 1–10: ANC-N, QBT-N, ANC-N, ANC-N, ANC-N, QMK-N, QMK-N, ANC-N, ANC-M, QMK-N. (JPG) [file pone.0323251.s004.JPG]

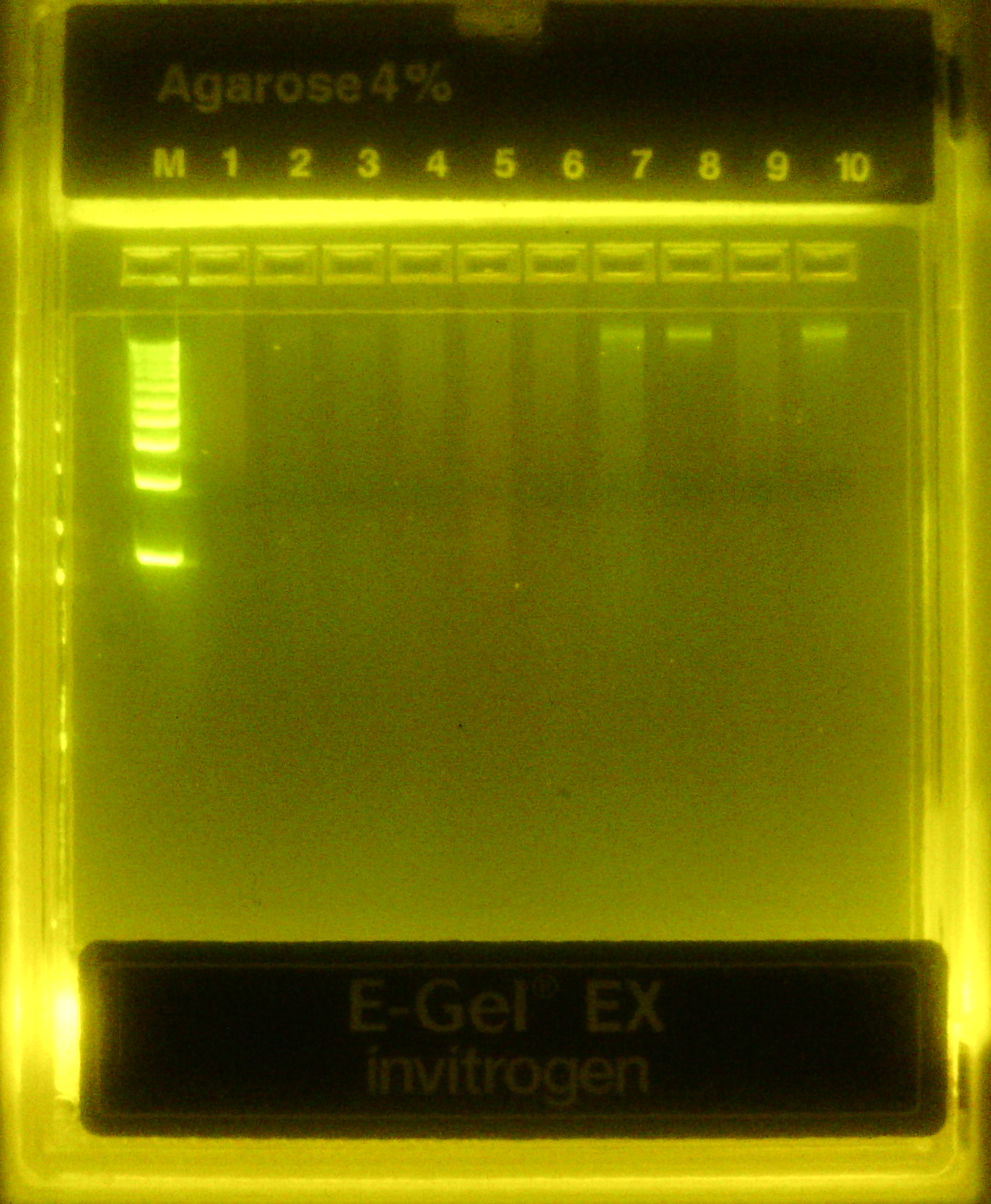

Supplement: S5 Fig — From well 1–10: AC-M, QBT-N, QBT-N, ANC-N, AC-N, ANC-N, QBT-N, QBT-N, ANC-N, QBT-N. (JPG) [file pone.0323251.s005.JPG]

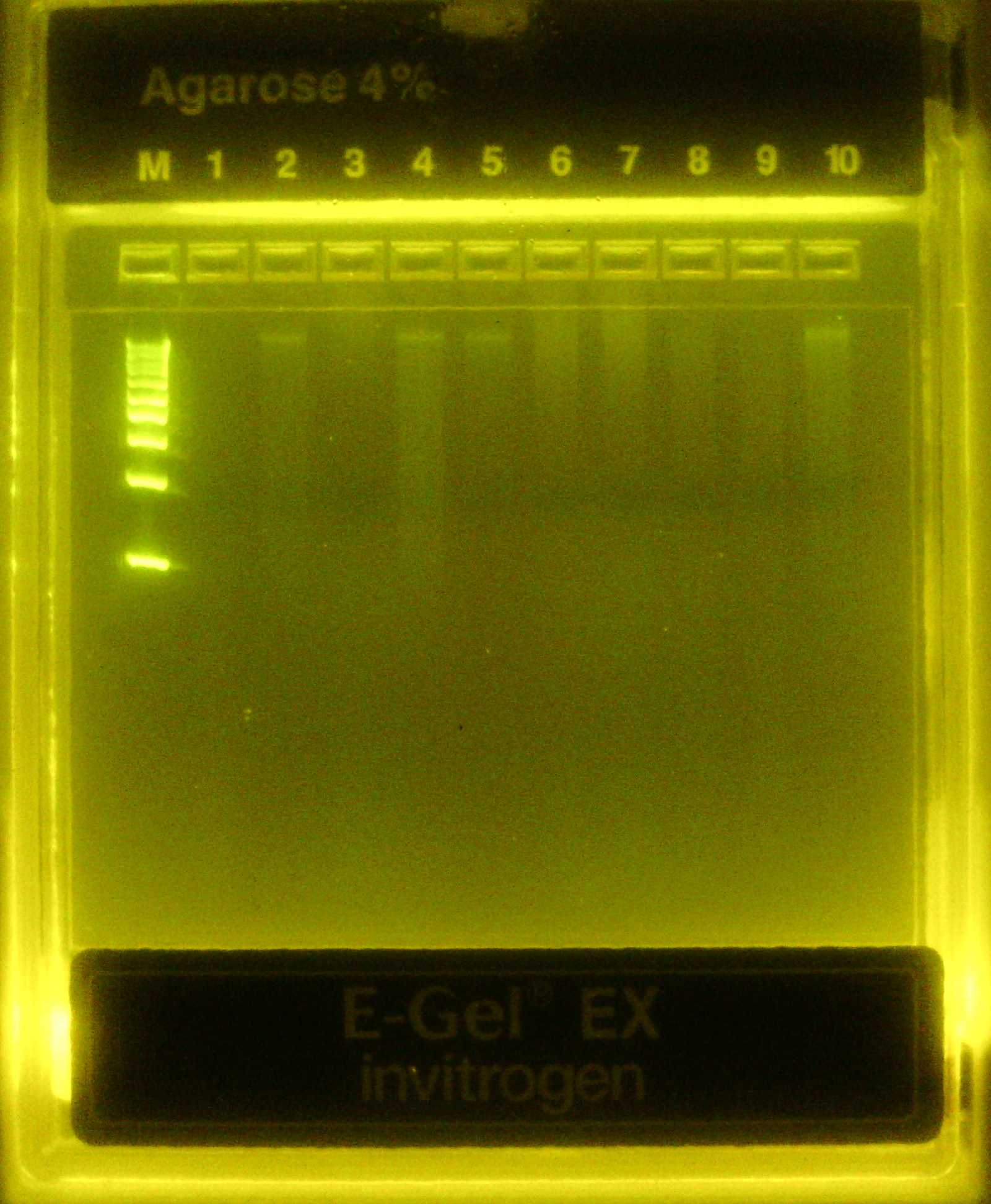

Supplement: S6 Fig — From well 1–10: ANC-F, QBT-N, ANC-N, QBT-F, QMK-N, ANC-N, AC-N, ANC-N, ANC-N, QMK-N. (JPG) [file pone.0323251.s006.JPG]

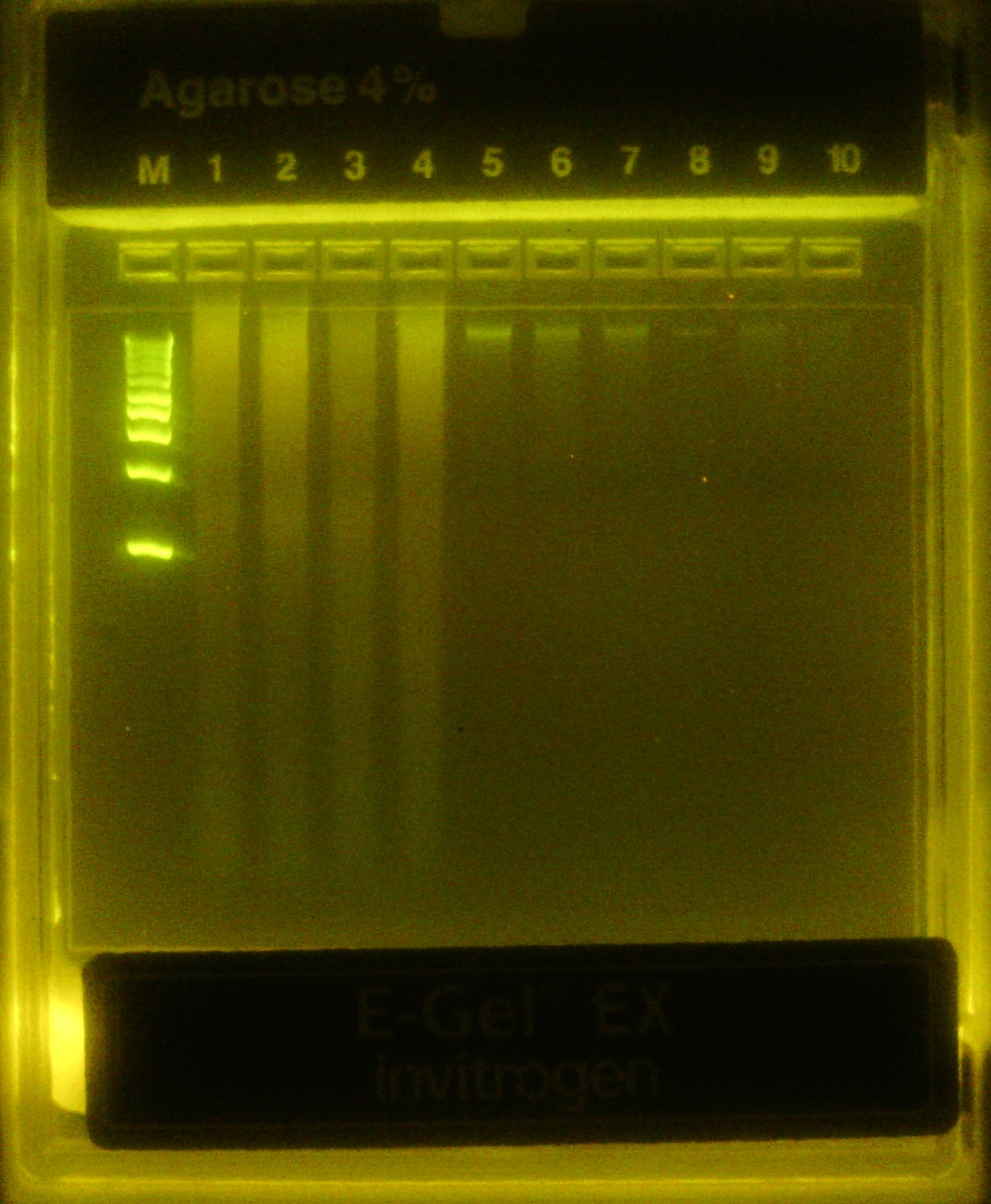

Supplement: S7 Fig — From well 1–10: AC-F, AC-F, ANC-F, ANC-F, QBT-N, QMK-N, QBT-N, QBT-N, QBT-N, QBT-N. (JPG) [file pone.0323251.s007.JPG]

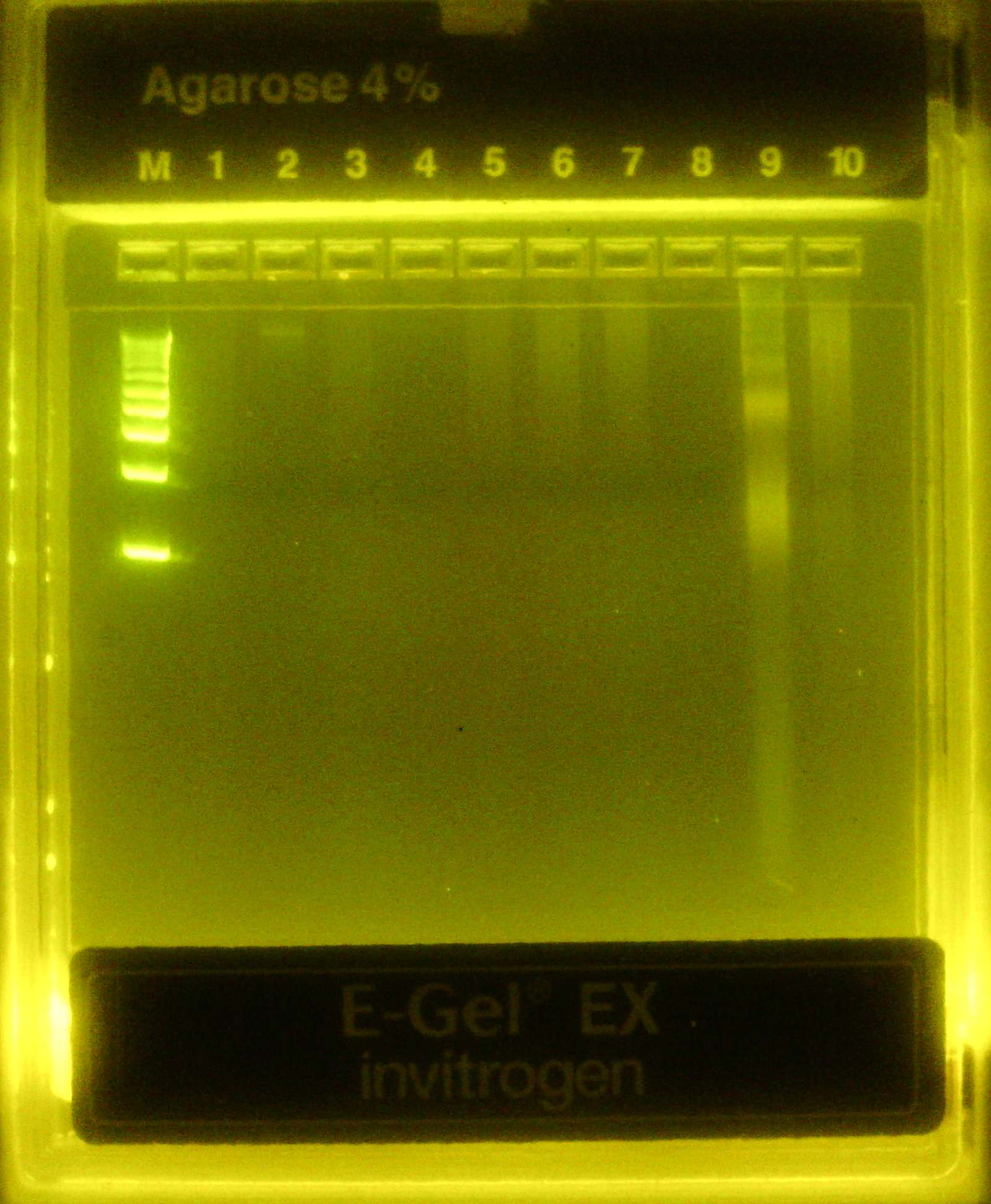

Supplement: S8 Fig — From well 1–10: ANC-N, QBT-N, ANC-N, QMK-N, ANC-N, ANC-N, AC-N, QBT-N, QBT-F, AC-N. (JPG) [file pone.0323251.s008.JPG]

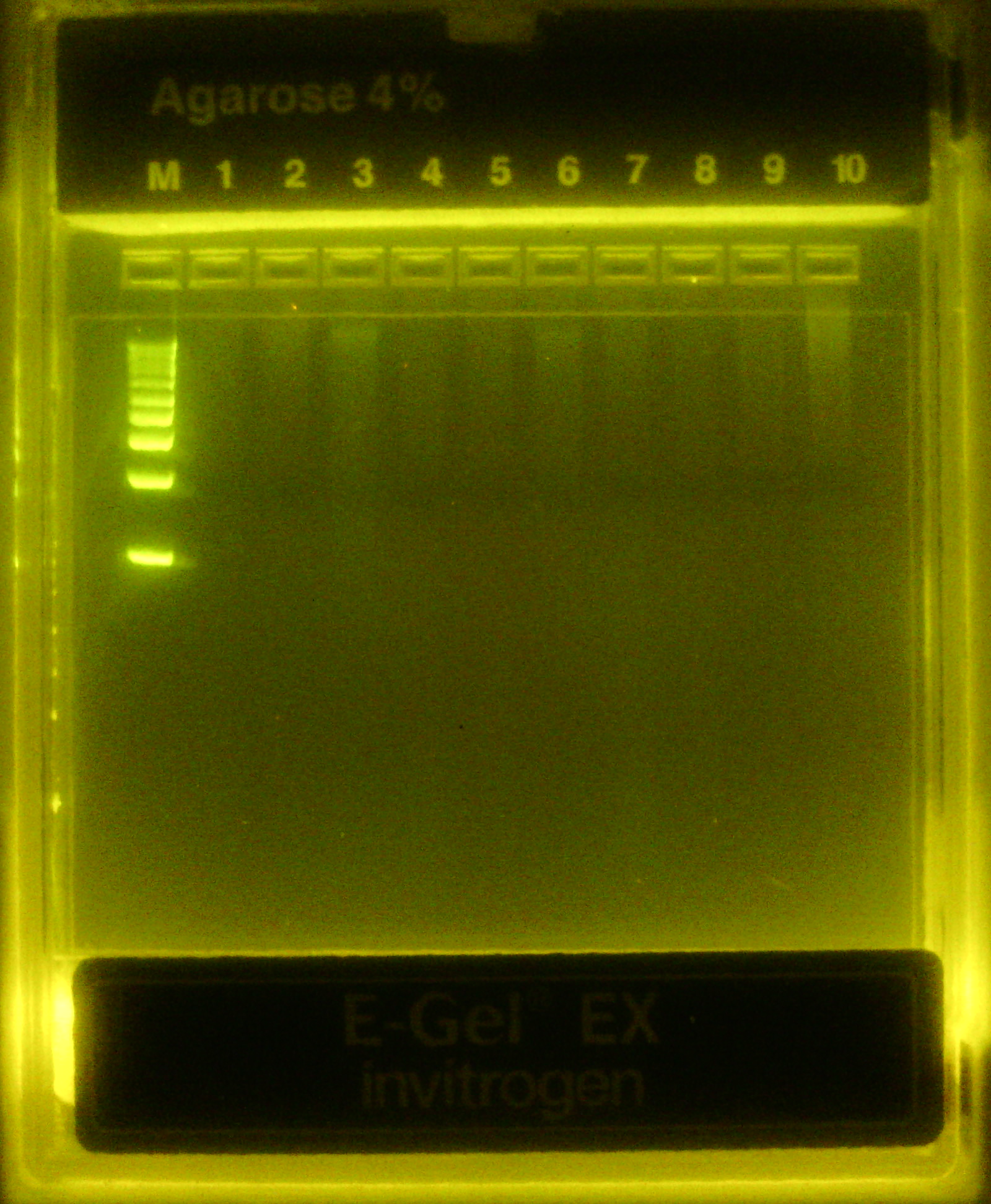

Supplement: S9 Fig — From well 1–10: ANC-N, AC-N, QMK-N, ANC-N, ANC-N, QBT-N, ANC-N, ANC-N, ANC-N, AC-N. (JPG) [file pone.0323251.s009.JPG]

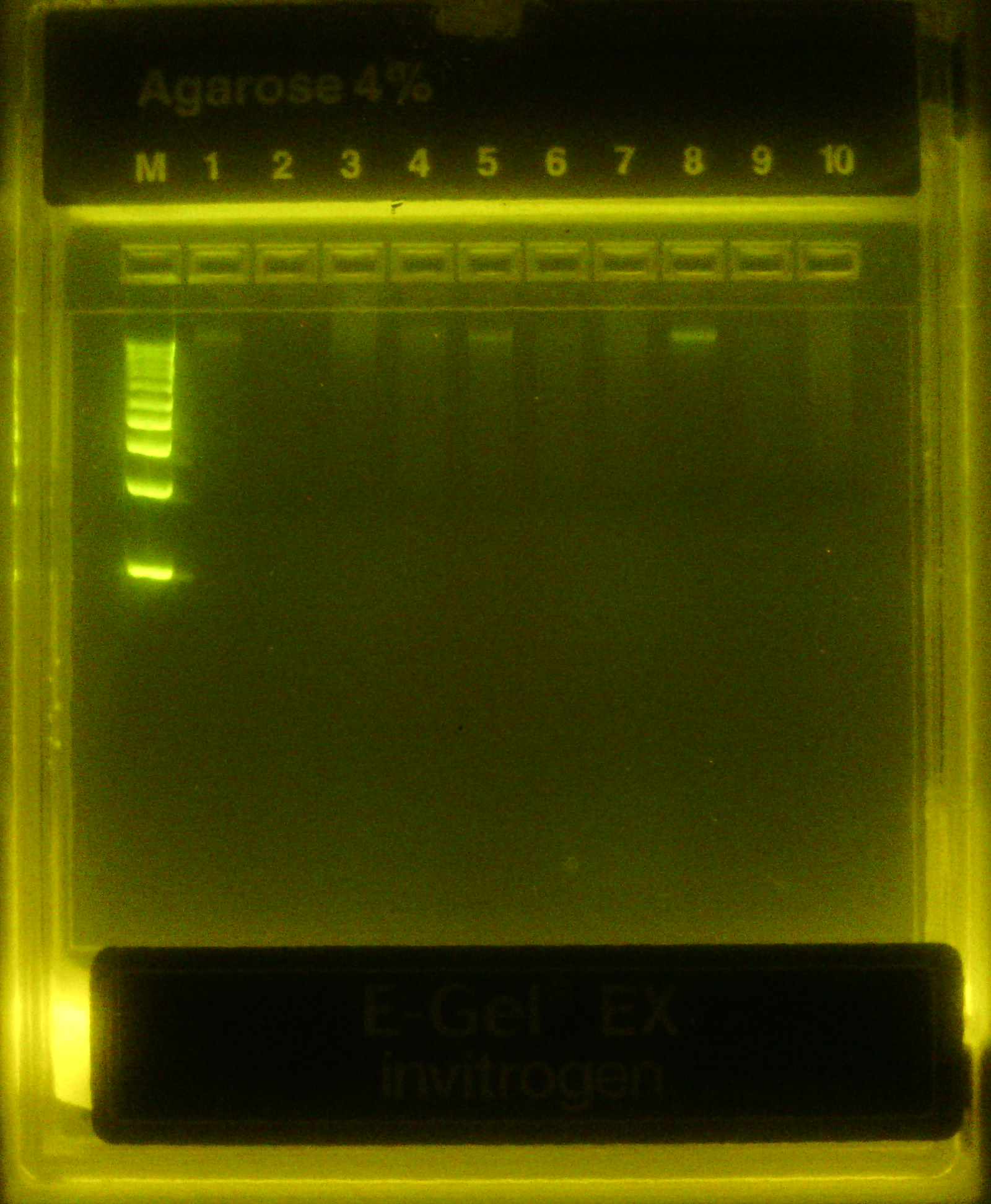

Supplement: S10 Fig — From well 1–10: QBT-N, ANC-N, AC-N, QMK-N, QMK-N, QBT-N, AC-N, QBT-N, QBT-N, AC-N. (JPG) [file pone.0323251.s010.JPG]

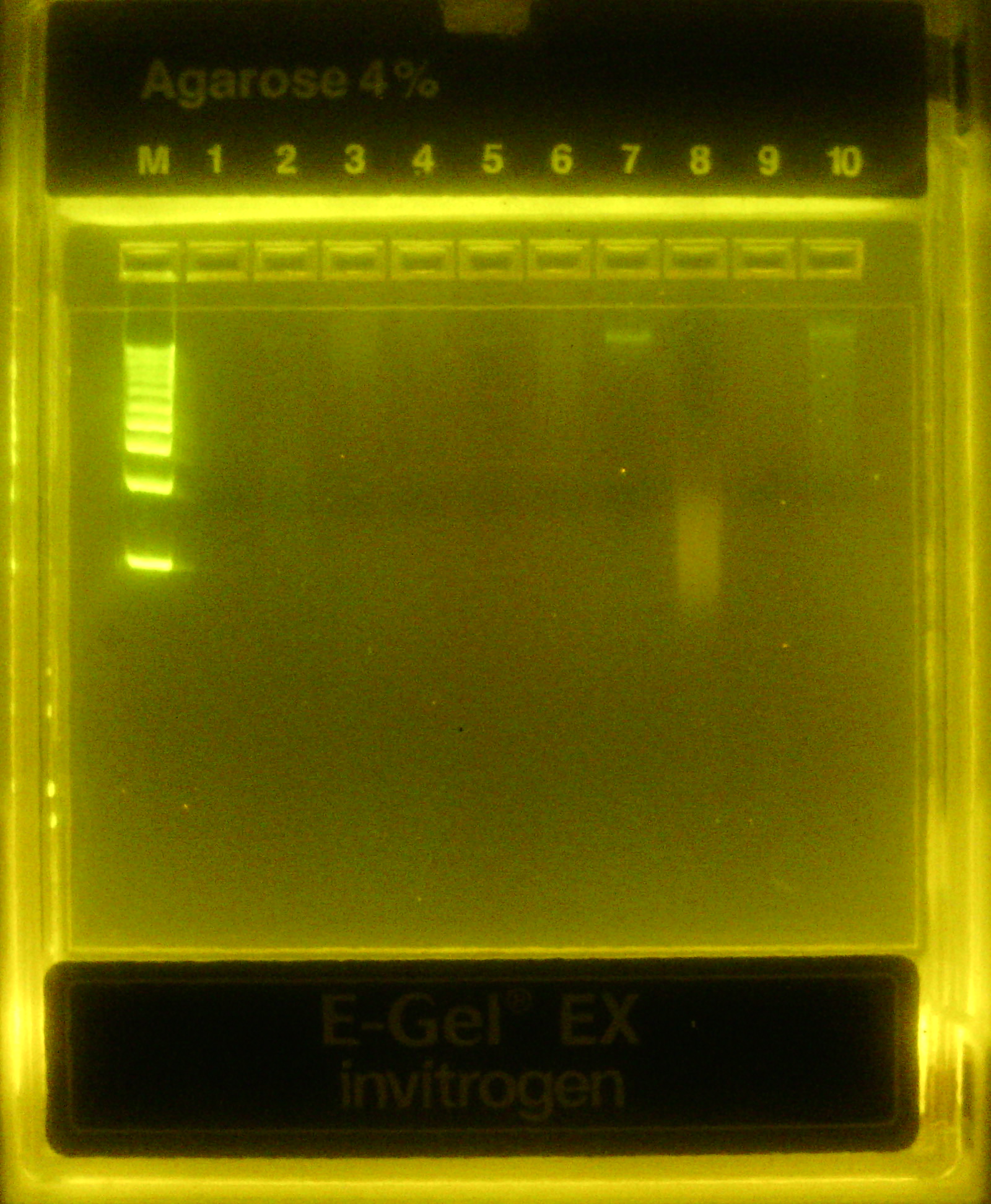

Supplement: S11 Fig — From well 1–10: ANC-N, QMK-N, AC-N, ANC-N, QMK-N, AC-N, QMK-N, AC-N, ANC-N, QMK-N. (JPG) [file pone.0323251.s011.JPG]

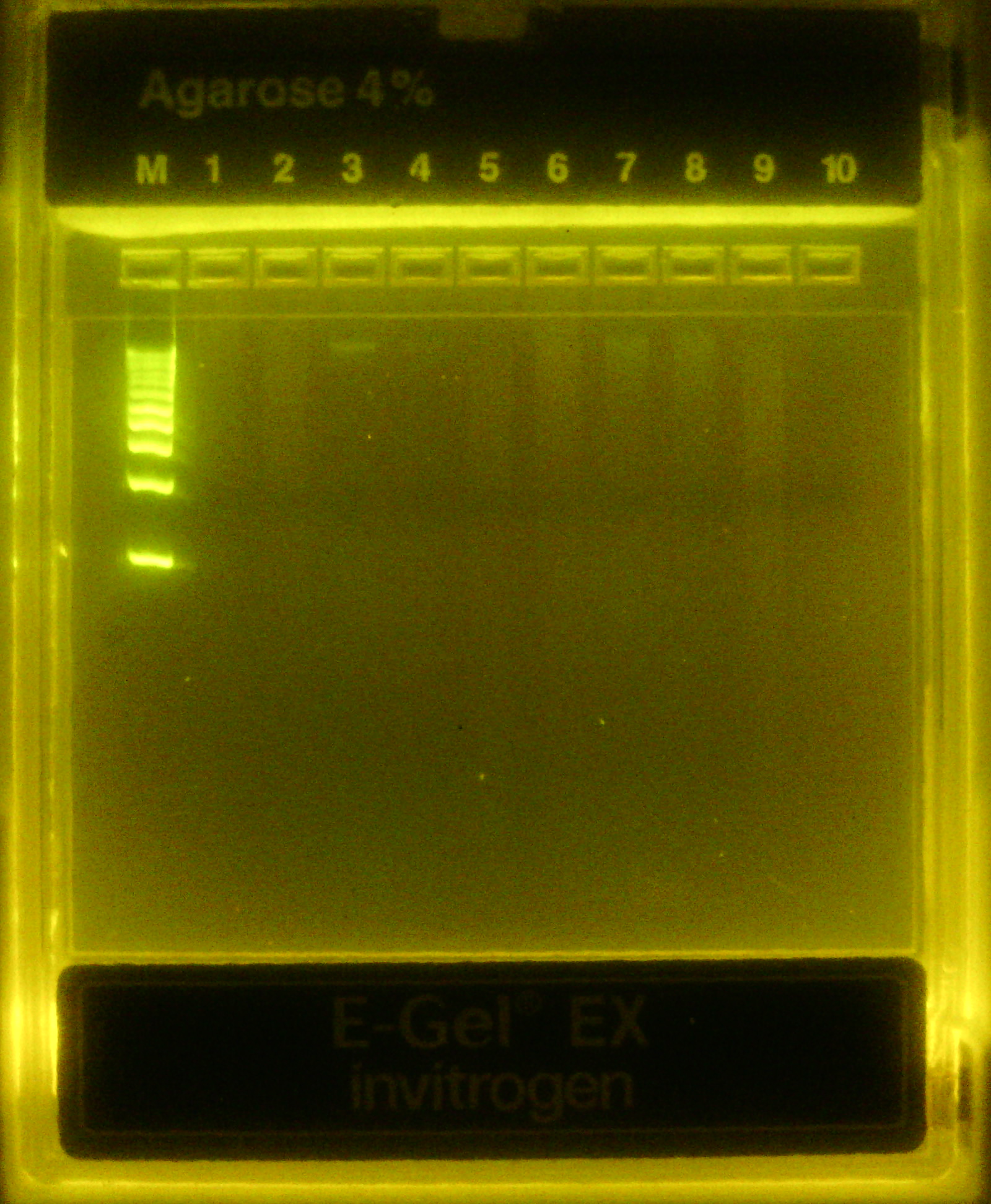

Supplement: S12 Fig — From well 1–10: AC-N, AC-N, QMK-N, QBT-N, ANC-N, AC-N, QBT-N, QMK-N, AC-N, QBT-N. (JPG) [file pone.0323251.s012.JPG]

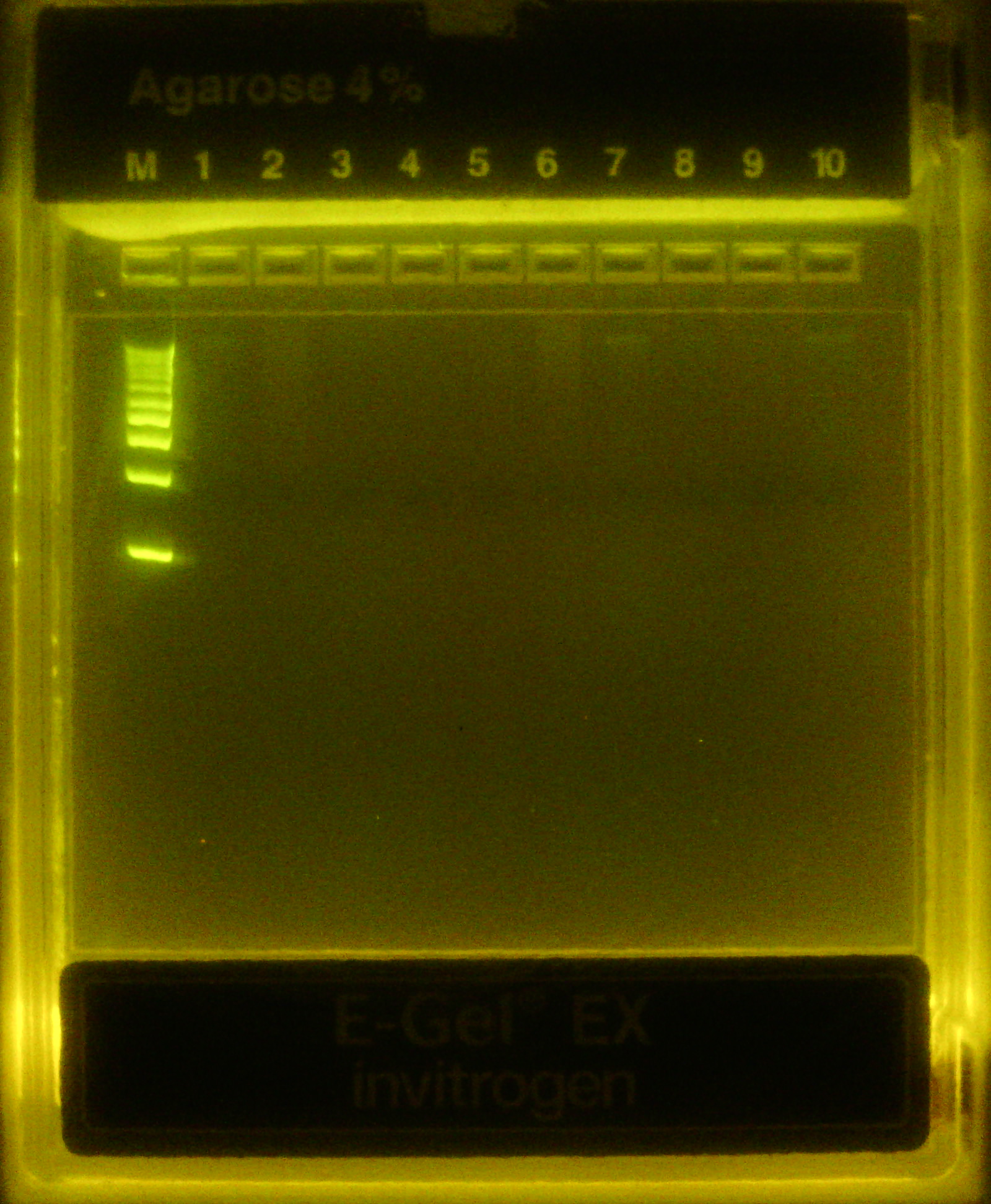

Supplement: S13 Fig — From well 1–10: QMK-N, AC-N, ANC-M, ANC-N, ANC-N, AC-N, QBT-N, QBT-N, ANC-N, QMK-F. (JPG) [file pone.0323251.s013.JPG]

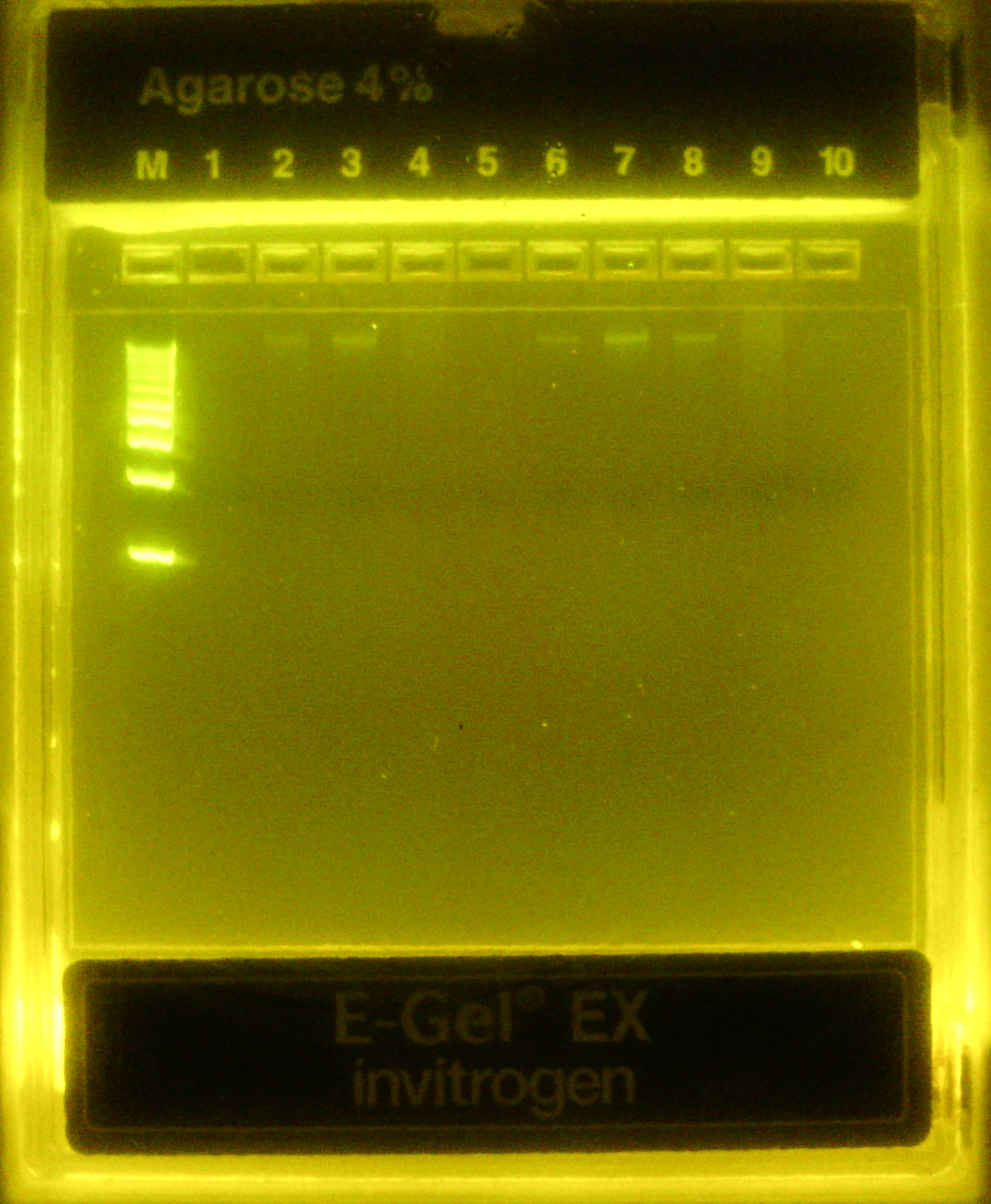

Supplement: S14 Fig — From well 1–10: QBT-N, QMK-N, QMK-F, AC-N, ANC-N, QMK-N, QMK-N, QBT-F, AC-N, QBT-N. (JPG) [file pone.0323251.s014.JPG]

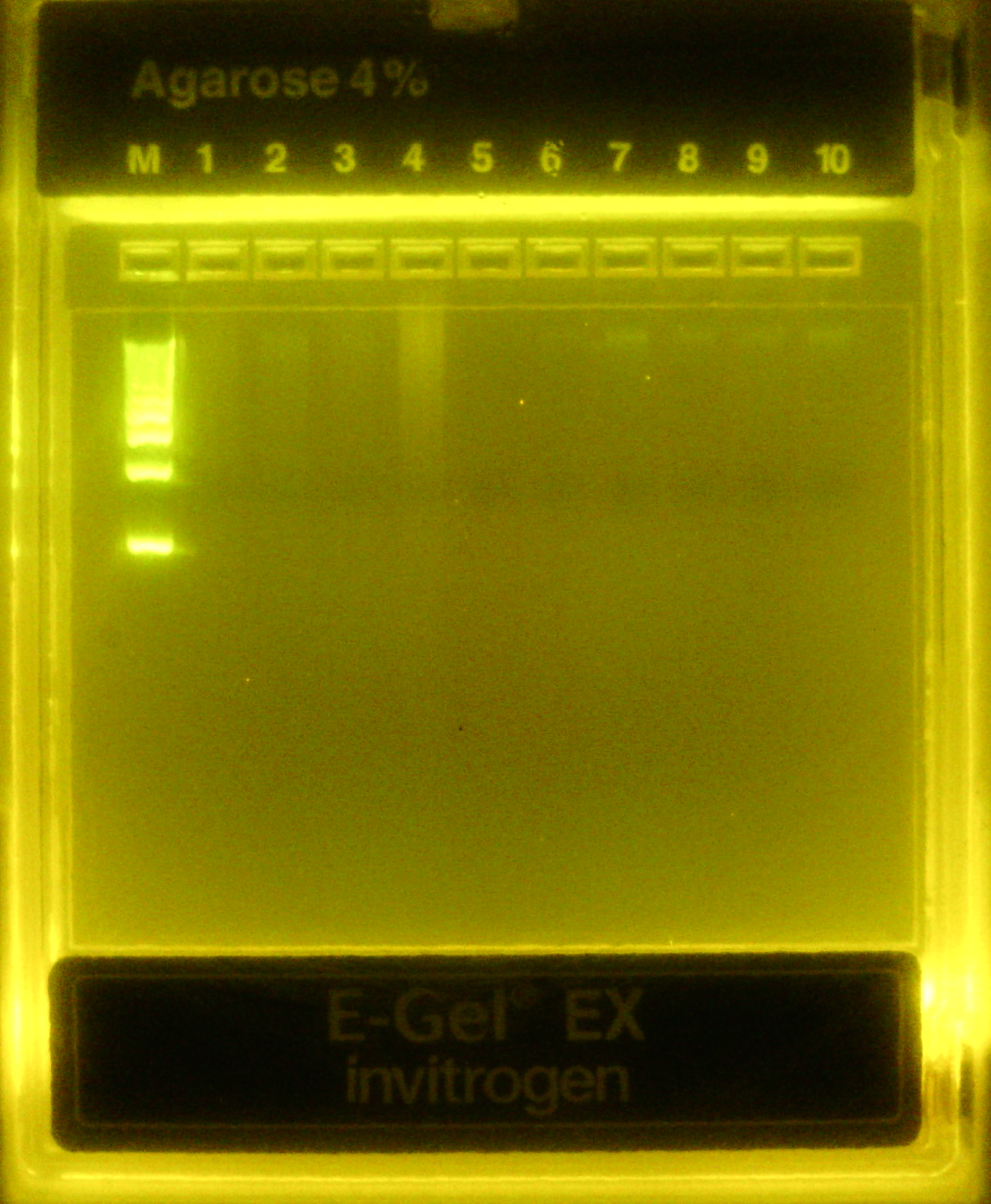

Supplement: S15 Fig — From well 1–10: AC-N, QBT-N, AC-N, AC-N, ANC-N, QBT-N, QMK-N, QBT-N, QMK-N, QBT-N. (JPG) [file pone.0323251.s015.JPG]

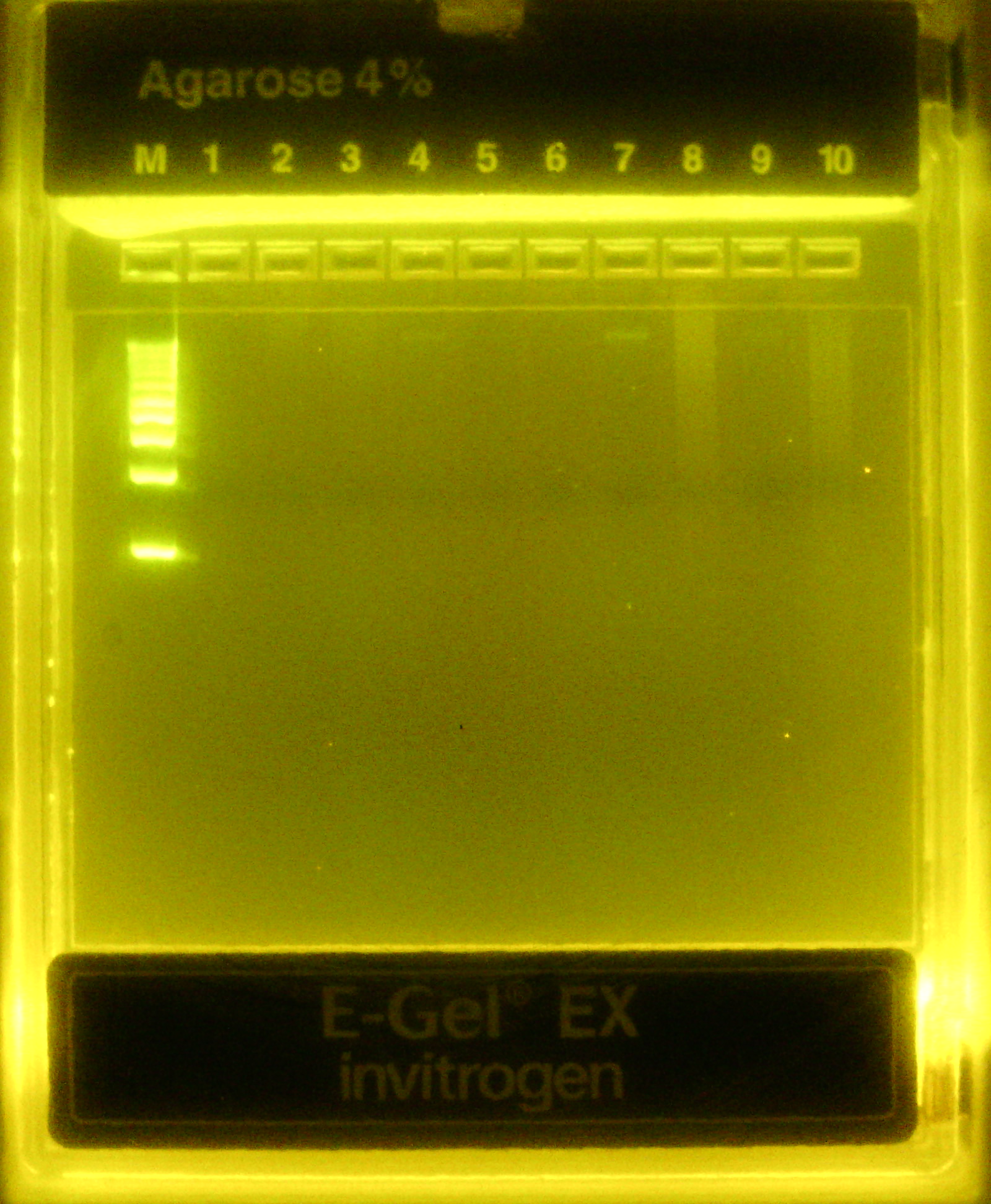

Supplement: S16 Fig — From well 1–10: ANC-N, AC-N, AC-N, QMK-N, QBT-N, ANC-N, QMK-N, AC-N, QMK-N, AC-N. (JPG) [file pone.0323251.s016.JPG]

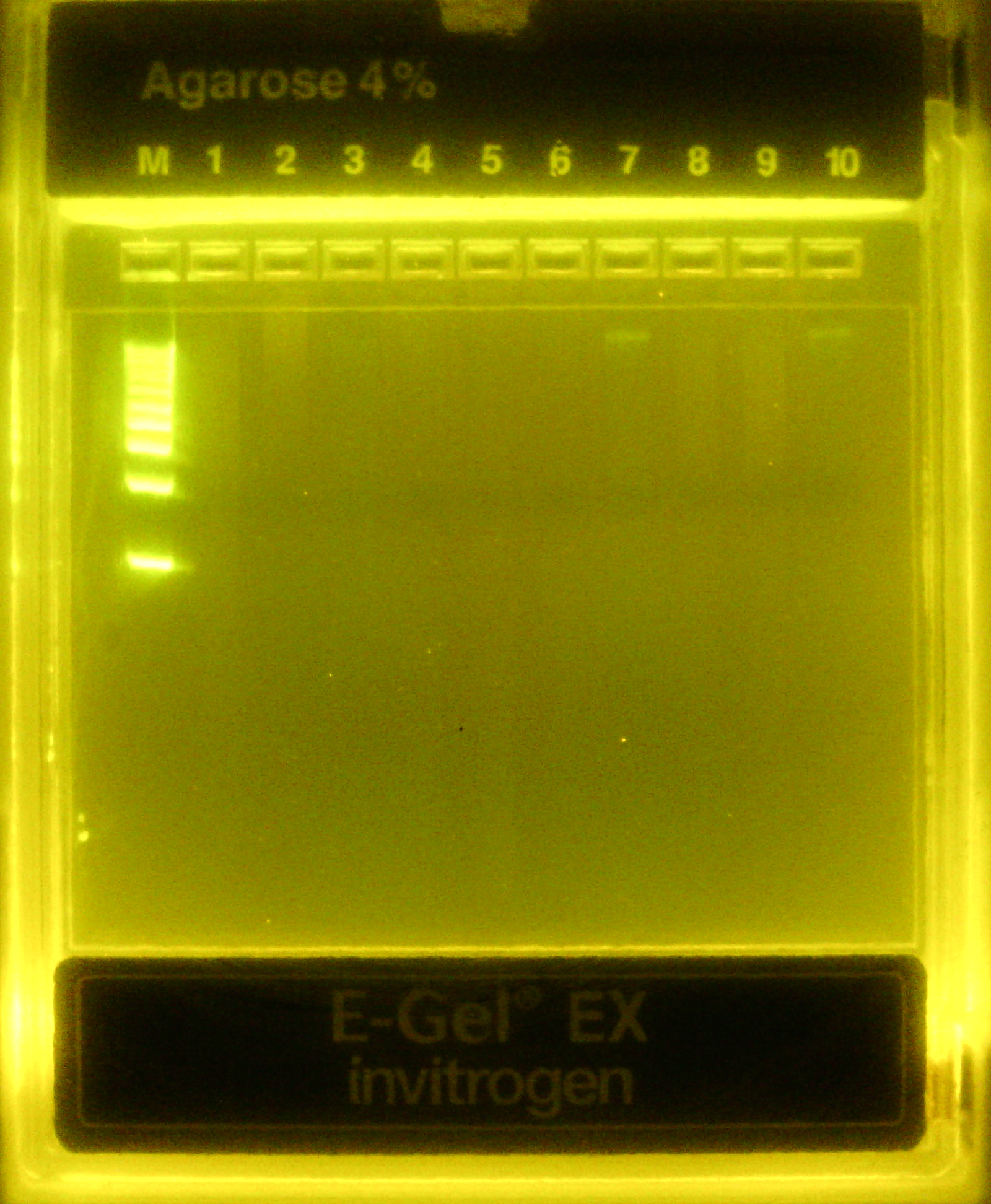

Supplement: S17 Fig — From well 1–10: AC-N, AC-N, QMK-F, AC-N, AC-M, AC-N, QMK-N, AC-N, AC-N, QMK-N. (JPG) [file pone.0323251.s017.JPG]

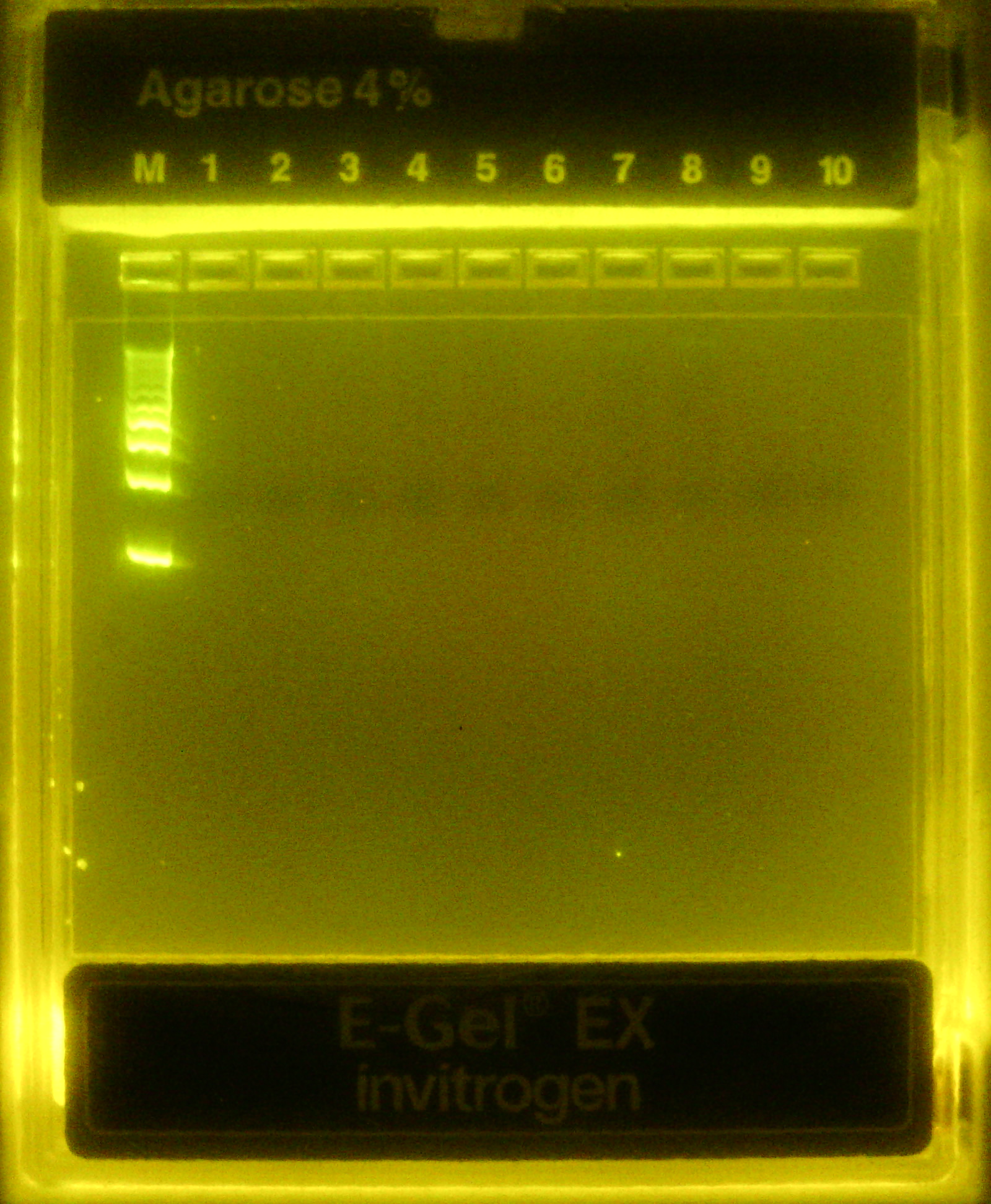

Supplement: S18 Fig — From well 1–10: AC-N, ANC-N, ANC-N, ANC-N, ANC-M, AC-N, QMK-N, QMK-N, AC-N, ANC-N. (JPG) [file pone.0323251.s018.JPG]

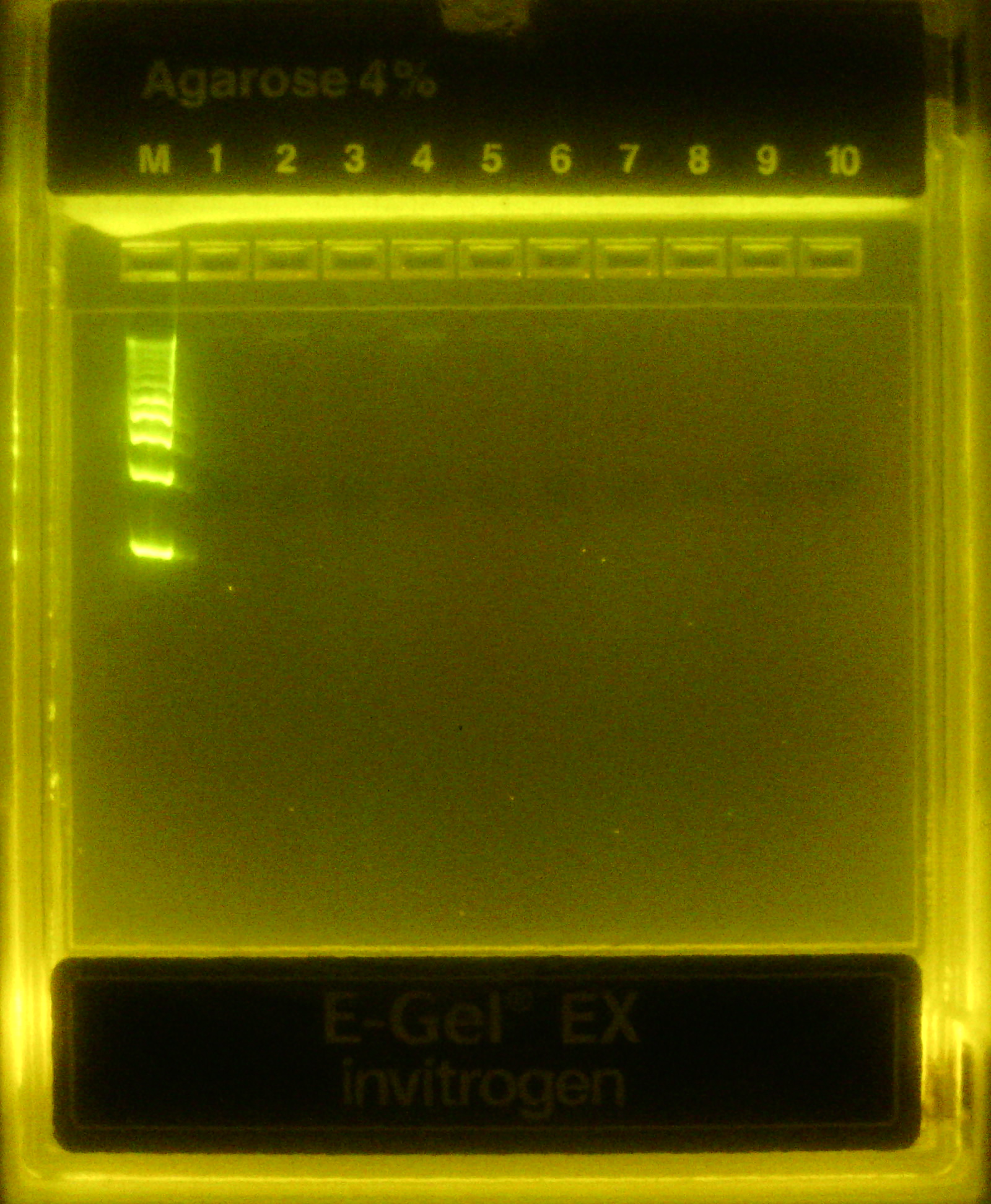

Supplement: S19 Fig — From well 1–10: QBT-N, QMK-N, QBT-N, QMK-N, QMK-N, QMK-N, AC-N, AC-N, QMK-M, QMK-N. (JPG) [file pone.0323251.s019.JPG]

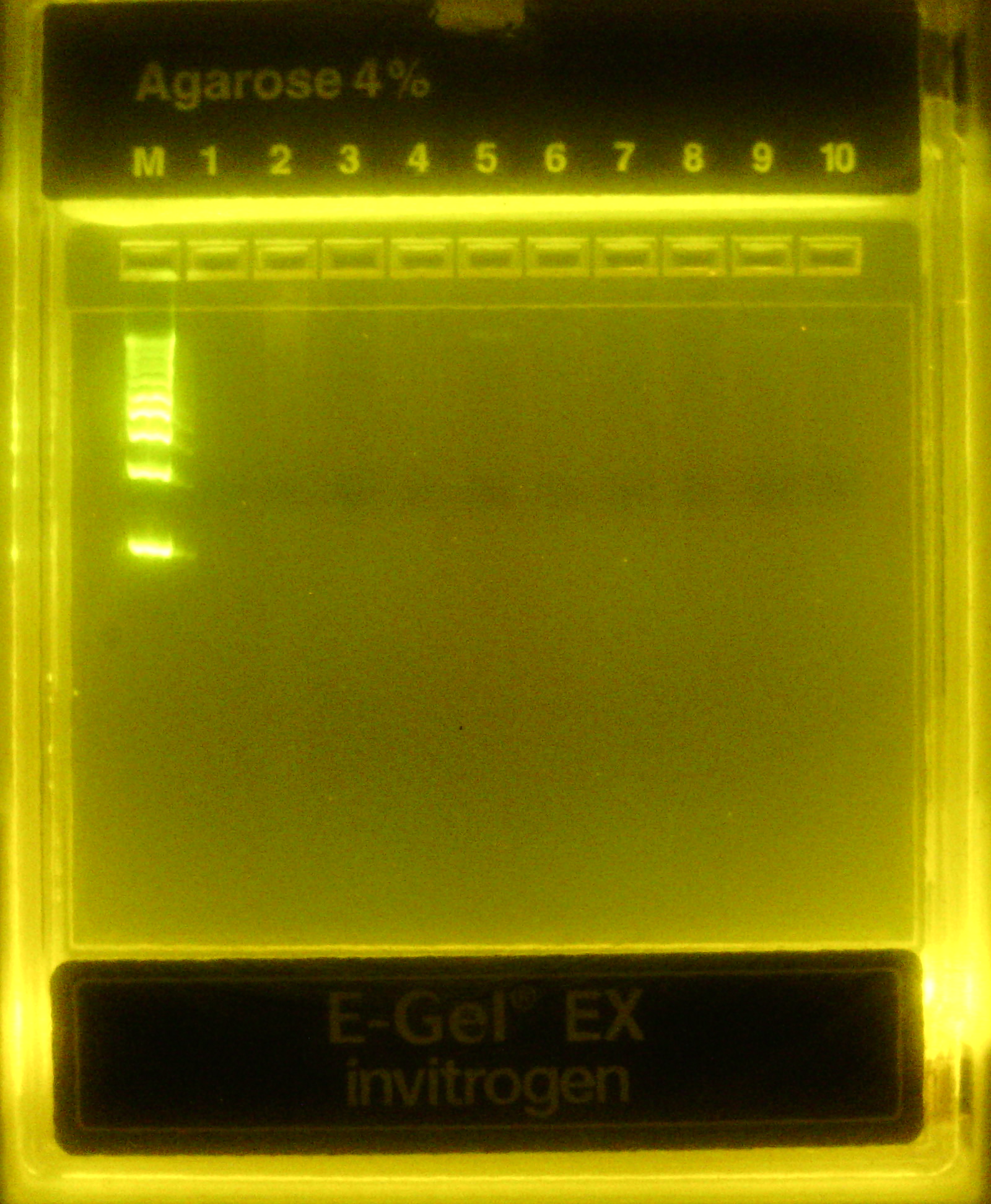

Supplement: S20 Fig — From well 1–10: QBT-M, AC-M, ANC-N, AC-N, QBT-N, QBT-N, QBT-N, QMK-M, QMK-M, QBT-M. (JPG) [file pone.0323251.s020.JPG]

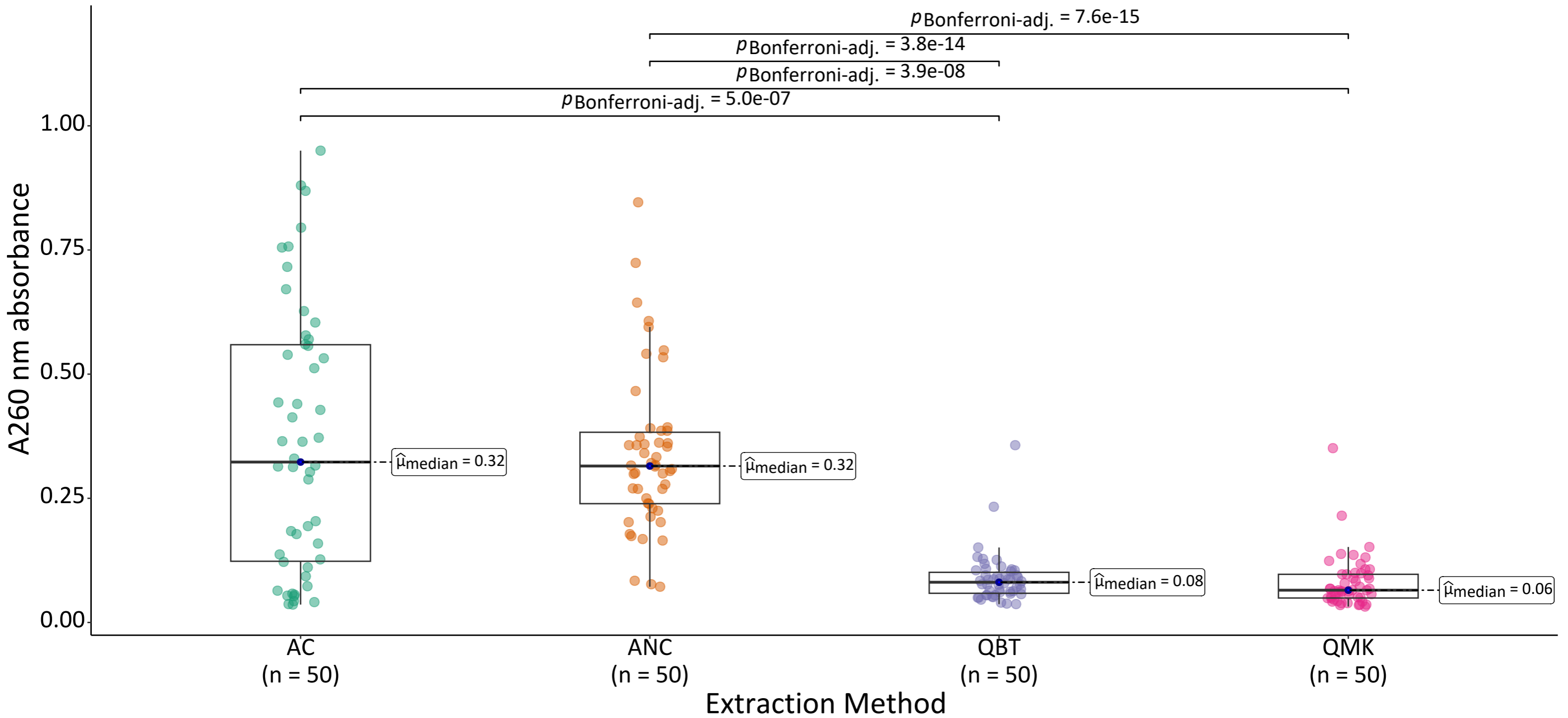

Supplement: S21 Fig — Results from the Kruskal-Wallis test are shown and significant (p < 0.05) differences in pair-wise comparison by Wilcoxon-Mann-Whitney with Bonferroni adjustment are shown at the top. (PDF) [file pone.0323251.s021.pdf]

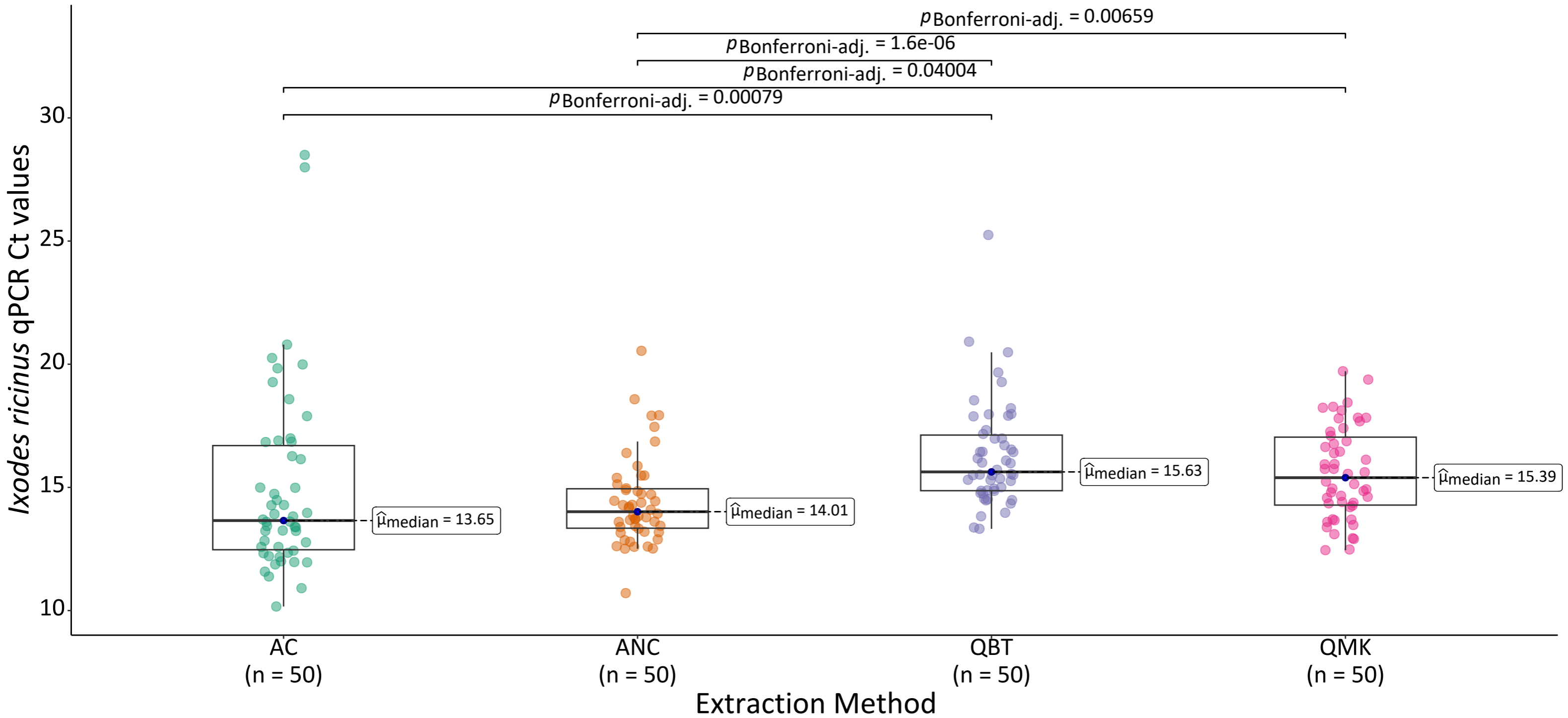

Supplement: S22 Fig — Results from the Kruskal-Wallis test are shown and significant (p < 0.05) differences in pair-wise comparison by Wilcoxon-Mann-Whitney with Bonferroni adjustment are shown at the top. (PDF) [file pone.0323251.s022.pdf]
