# Supplementary material for: Making the best of a bad sample: Comparison of DNA extraction and quantification methods using sub-optimally stored Ixodes ricinus ticks
Source: PLoS One. 2025 May 29;20(5):e0323251. doi: 10.1371/journal.pone.0323251 (PMC12121741; doi:10.1371/journal.pone.0323251)
Supplement: S1 Table — (DOCX) [file pone.0323251.s023.docx]

S1 Table. Overall results from the four extraction methods

| Measurement | Stage/Sex | Ammonium hydroxide | | | | | | | | Qiagen DNA extraction kit | | | | | | | |
| --- | --- | --- | --- | --- | --- | --- | --- | --- | --- | --- | --- | --- | --- | --- | --- | --- | --- |
|  |  | Not homogenized (ANC) | | | | Pre-homogenized (AC) | | | | Mini Kit (QMK) | | | | Blood & Tissue (QBT) | | | |
|  |  | Min | Max | Avg | Med | Min | Max | Avg | Med | Min | Max | Avg | Med | Min | Max | Avg | Med |
| NanoDrop DS  (ng) | N | 820 | 3800 | 1788 | 1590 | 280 | 9700 | 2689.8 | 2580 | 320 | 2160 | 759.5 | 640 | 380 | 3560 | 888.9 | 780 |
|  | ♂ | 1520 | 4200 | 2796.7 | 2670 | 890 | 10200 | 5263.3 | 4700 | 560 | 3520 | 1600 | 720 | 380 | 940 | 660 | 660 |
|  | ♀ | 4230 | 19700 | 12610 | 13900 | 3780 | 7900 | 5726.7 | 5500 | 400 | 1080 | 720.0 | 680 | 500 | 1320 | 966.7 | 1080 |
| NanoDrop SS (ng) | N | 170 | 2170 | 1050.9 | 1005 | 100 | 3580 | 1196.1 | 1075 | 40 | 2200 | 638.6 | 530 | 140 | 3280 | 709.3 | 640 |
|  | ♂ | 280 | 1700 | 1006.7 | 1040 | 290 | 1030 | 673.3 | 700 | 120 | 326 | 222 | 220 | 60 | 400 | 230 | 230 |
|  | ♀ | 780 | 2430 | 1390 | 960 | 260 | 2720 | 1270 | 830 | 60 | 400 | 233.3 | 240 | 140 | 680 | 406.7 | 400 |
| Qubit  (ng) | N | 12.2 | 218 | 98.8 | 85.7 | 12.2 | 142 | 53.1 | 48.2 | 28 | 740 | 185.7 | 127 | 24 | 788 | 266 | 200 |
|  | ♂ | 23 | 150 | 76.3 | 55.8 | 12.4 | 147 | 62.9 | 29.2 | 28.8* | | | | 27.2* | | | |
|  | ♀ | 131 | 736 | 525 | 708 | 195 | 1260 | 743.7 | 776 | 66.8 | 105.2 | 91.7 | 103.2 | 96.8 | 246 | 177.1 | 188.4 |
| Ixodes Ct | N | 12.5 | 20.5 | 14.4 | 13.9 | 10.9 | 28.5 | 15.2 | 13.8 | 12.5 | 19.7 | 15.5 | 15.4 | 13.3 | 25.2 | 16.3 | 15.7 |
|  | ♂ | 14.5 | 17.9 | 15.8 | 14.9 | 13.4 | 16.8 | 14.8 | 14.3 | 16.8 | 19.4 | 17.8 | 17.4 | 16.7 | 18.5 | 17.6 | 17.6 |
|  | ♀ | 10.7 | 15.1 | 12.8 | 12.6 | 10.2 | 12.4 | 11.5 | 12 | 14.4 | 14.9 | 14.6 | 14.6 | 14.5 | 14.9 | 14.8 | 14.9 |
| NM Ct | N | 18.2 | 19.8 | 18.7 | 18.6 | 18.2 | 25 | 19.4 | 18.9 | 18.3 | 19.4 | 18.7 | 18.7 | 18.4 | 19.7 | 18.8 | 18.7 |
|  | ♂ | 18.8 | 19 | 18.9 | 19 | 18.8 | 23.4 | 20.4 | 19.2 | 18.6 | 19.4 | 19 | 18.8 | 18.7 | 19 | 18.8 | 18.8 |
|  | ♀ | 18.5 | 19.4 | 19.1 | 19.3 | 18.9 | 19.8 | 19.5 | 19.8 | 18.6 | 19 | 18.7 | 18.7 | 18.7 | 19.8 | 19.1 | 18.7 |
| A260 | N | 0,07 | 0,72 | 0,33 | 0,32 | 0,04 | 0,95 | 0,38 | 0,36 | 0,03 | 0,22 | 0,08 | 0,06 | 0,04 | 0,36 | 0,09 | 0,08 |
|  | ♂ | 0,08 | 0,53 | 0,31 | 0,31 | 0,09 | 0,20 | 0,16 | 0,18 | 0,06 | 0,35 | 0,16 | 0,07 | 0,04 | 0,10 | 0,07 | 0,07 |
|  | ♀ | 0,28 | 0,85 | 0,51 | 0,39 | 0,11 | 0,76 | 0,34 | 0,16 | 0,04 | 0,11 | 0,07 | 0,07 | 0,05 | 0,13 | 0,10 | 0,11 |
| A260/280 | N | 1.1 | 1.7 | 1.5 | 1.6 | 0.4 | 1.7 | 1.4 | 1.4 | 0.3 | 2.6 | 1.4 | 1.4 | 1.3 | 2.3 | 1.6 | 1.6 |
|  | ♂ | 1.2 | 1.4 | 1.3 | 1.4 | 1.3 | 1.5 | 1.4 | 1.4 | 1.0 | 1.2 | 1.1 | 1.2 | 1.4 | 1.8 | 1.6 | 1.6 |
|  | ♀ | 1.4 | 1.5 | 1.5 | 1.5 | 1.4 | 1.5 | 1.4 | 1.5 | 1.4 | 1.7 | 1.5 | 1.4 | 1.6 | 2.0 | 1.8 | 1.8 |

N: Nymphs; ♂: males; ♀: females; DS: doble stranded; SS: single stranded; NM: spiked samples with *Neoehrlichia mikurensis* DNA; Avg: average; Med: median; * only one sample was measurable for the Qubit.
