# Supplementary material for: Making the best of a bad sample: Comparison of DNA extraction and quantification methods using sub-optimally stored Ixodes ricinus ticks
Source: PLoS One. 2025 May 29;20(5):e0323251. doi: 10.1371/journal.pone.0323251 (PMC12121741; doi:10.1371/journal.pone.0323251)
Supplement: Attachment — Submitted filename: Comments to the Authors.docx [file pone.0323251.s025.docx]

**Comments to the Authors**

Cotes-Perdomo et al: Making the best of a bad sample: Comparison of DNA extraction and quantification methods using sub-optimally stored *Ixodes ricinus* ticks

**Major Comments**

Cotes-Perdomo et al evaluate different methods of DNA extraction and quantification of *Ixodes ricinus* ticks that were stored for over 10 years at room temperature in 100% ethanol, which had evaporated from several samples prior to extraction. The authors tested ammonia hydrolysis methods (with homogenized ticks and non-homogenized ticks) and two industry standard kits from Qiagen (with homogenized ticks) to extract DNA. They also tested different DNA quantification methods (qPCR, fluorometry, drop spectrophotometry, gel electrophoresis) after DNA extraction to determine the best methods to measure quality and quantity. The authors found that extracting DNA from whole ticks using the ammonia hydrolysis method was not only better at extracting more DNA, but was also more cost-effective and less labor-intensive than the other methods. Further, qPCR was the best method to quantify DNA.

Overall, the authors provide good information on a question that likely many authors in the tick world have had about the viability of samples, especially if resources for DNA extraction and/or testing are not readily available. To improve the manuscript, there are several major changes and clarifications that should take place before the paper is ready for publication.

One major concern with the paper is that the results are not separated by tick life stage. The authors tested nymphal, female, and male ticks, all of which may have different amounts of DNA present. This could potentially affect the statistics of the study and thusly, interpretation of the results. If the authors chose to combine the life stages during the data analysis phase due to no significant difference in the amount of DNA present across the different life stages and the data were not shown, then they should mention this circumstance and provide the data either in the manuscript or as supplementary materials. Separating the results based on life stage would be more impactful as many researchers often selectively test different life stages due to resource availability, pathogen presence in certain life stages, etc.

Furthermore, the methods need additional clarifying details, especially the statistical analyses. The authors reported results for which no methods were mentioned or the methods did not provide enough detail to clearly interpret the results. For example, the authors did not add information on the ratios they calculated to compare the DNA extraction and measurement methods; the results were explained in lines 266-274 and table 5. More specifically, what values were used to calculate the ratios? In addition, their GLM also needs more context, including information on the covariates that went into the model and the outcome variable as well as the link function and distributions used. It is difficult to interpret the results mentioned in lines 194-196 and 291-294 without the context of what went into the model. The authors also conducted a correlation analysis and produced correlation coefficients to compare the DNA measurement across the DNA extraction methods (see lines 279-290, table 6), but this was not mentioned anywhere in the methods. Information on how the coefficients were calculated (what values were used?) and how the authors interpreted the results would be helpful. While some of the methods were provided in the figure caption, the information was not in the actual main manuscript text. With a lot of missing information from the methods, the results are difficult to interpret.

Furthermore, there was no information on the role of testing for *N. mikurensis* and *B. burgdorferi* in the study. While *N. mikurensis* was used to detect PCR inhibition, this was not the case for *B. burgdorferi*. It’s reasonable for the authors to include those results in the manuscript, but that should be clearly stated as a new objective in the study because testing for those two pathogens seemed out of place when that was not the main purpose of this study. If the authors choose to keep this information in the manuscript, they need to include the significance of these pathogens in the introduction and/or discussion to justify reporting the results in the manuscript.

Finally, the figures and tables could be improved by providing more context in the captions and/or the manuscript text. Similar to what was said previously about results reporting, it would help readers if the authors included how the data in the figures/tables were calculated, where necessary. Figure 2 doesn’t really add much to the manuscript. Instead, I suggest having a figure show the different shapes described in lines 239-252. Figure 3 could be improved if the authors included all of the DNA quantification methods in one figure to compare DNA yields per DNA extraction and quantification method. That is, take supplementary figures 21-23 and make a multi-panel figure so that readers can compare the quantities directly rather than having to pull up the supplementary figures.

These are a few of the major changes I think the authors should consider before resubmitting the manuscript. The manuscript has value, but the authors should highlight the most important information in the main text, figures, and tables and provide substantial clarity to their methods such that their results could be more accurately interpreted. More specific line-by-line comments are provided below.

**Line-by-Line Comments**

Abstract: I would check the submission instructions for the journal, but the abstract should probably be one large paragraph rather than separate paragraphs.

Lines 15-19: It would be helpful to briefly include how performance was evaluated for each DNA extraction method and how yield and purity were evaluated. This would provide more context overall to the rest of the abstract.

Line 16: Which life stages of ticks were tested?

Line 20 (and elsewhere): There are several instances where the authors use adverbs that may not necessarily be useful in the sentence’s context and/or cannot be quantified. In this case, “very” does not need to be included. The authors should go through and remove these adverbs from the manuscript accordingly.

Line 25 (and elsewhere): “… drop spectrophotometry…”

Line 26: What do you mean by “correlated poorly?” What values were compared for the correlation and how were the correlations calculated?

Lines 28-29: Which methods were used to detect *N. mikurensis* and *B. burgdorferi*?

Introduction

Line 60: “… hydrolysis method (11-13) has the advantage…”

Line 60 (and elsewhere): Is it “ammonia hydroxide hydrolysis” or “ammonia hydrolysis?” There are several instances where either of the terms are used, but it would be better to stick with one term and to use it consistently throughout the manuscript.

Line 71: “… for pure double-stranded (DS) or single-stranded (SS) DNA, and corrections…”

Lines 70-78: The sentences need to be restructured as they are quite long-winded and confusing to read. I suggest something like this – “While this method is quite accurate for pure double-stranded (DS) or single-stranded (SS) DNA and corrections for the presence of moderate amounts of protein are available, A260 will not be an accurate measure of DNA concentration if the sample contains a mixture of DS and SS DNA (whose A260s differ) or if other substances containing aromatic rings, such as ATP, RNA, and phenol are present. Further, the A260:280 ratio is not a foolproof measure of molecular purity as many impurities have no absorbance in this region. It would be possible to construct a mixture of aromatic compounds having an A260:280 ratio of 1.8 while containing no DNA at all, for example.” This is also where you have the first mention of double-stranded and single-stranded DNA, so only the abbreviations can now be used throughout the rest of the manuscript.

Lines 101-102: “… two related commercial DNA extraction kits that use silica membranes…”

Line 104 (and 392): It would be helpful to explicitly define “sub-optimal” conditions either here in this line, methods, or both. This was alluded to earlier in the introduction (lines 44-47), but it should be more clearly defined.

Methods

Line 113: Could ticks still submerged in ethanol have more DNA preserved? Did any samples have ticks still submerged in ethanol when it was time to conduct DNA extractions or were all considered dry? If submerged ticks were used, this could have affected the results of the DNA extraction and quantities. Please provide more information, maybe an approximate percentage of ticks that were still in ethanol prior to the extractions.

Lines 121-131: Please provide citations for the extraction methods using ammonia.

Line 139 (and elsewhere): Should “concentration” be replaced with “quantity?” In most cases, concentrations are not the same as quantities.

Lines 139-177: The order of the methods to quantify and evaluate DNA should be the same order in which they are introduced in the introduction. Similarly, the results should follow a consistent order as well, where appropriate. In addition, please provide the amounts of extracted DNA used for each DNA quantification method.

Lines 140-142: Please add more information on how the gel electrophoresis was run. Include information such as the voltages used, amount of sample used per well (see previous comment) and time used to run the gels.

Lines 163-165 and Tables 2 and 3: Tables should be switched, where the table of primers is listed first and then the PCR settings. In addition, a brief description about *5.8s* rRNA should be provided (what is it? What does it control?). A reference from the literature describing *5.8S* rRNA, its primers, and protocol should also be provided in the main text and not just in the table.

Lines 172-175: “… as an anomalous amplification curve.” Also, please provide a reference for this sentence.

Lines 178-186: This section seems very out of place compared to the rest of the manuscript. I understand the purpose of including *N. mikurensis* as a way to test for PCR inhibition, but then why include *B. burgdorferi* if that was not going to be used in the same way as *N. mikurensis*?

Line 183: Were all samples spiked with pNeo? Or just some? If only some were spiked, how were they selected?

Lines 183-186: Can you provide more information on PCR inhibition and how it relates to sample spiking in the introduction and/or briefly in the methods?

Line 186: Remove the underline from the period at the end of the sentence.

Lines 188-193: The way the sentence is currently written, it sounds like you are comparing the listed measurements to each other rather than comparing the measurements of the different extraction methods. If the latter is the case, please rephrase the sentence for clarity. In addition, “results are presented in Supplementary Table 1 and corresponding figures” can be deleted. This can be mentioned in the actual results section. Furthermore, supplementary table 1 can probably be moved to the main text as this is interesting information. If it’s moved to the main text, you should bold or add notations that mark significant differences.

Line 194: “A generalized linear model…”

Lines 194-196: This sentence does not make sense. More information on the GLM needs to be provided in general, see major comments.

Line 198: R was used to do the statistics, but please also provide R packages that were used to calculate any measurements or produce data. In addition, information on Excel was not provided here even though a function used in Excel was mentioned in a table to calculate correlation coefficients (lines 288-289). Information on how the correlation coefficients were calculated were also not provided (what was the equation and what values were used?), but they need to be mentioned somewhere in the methods, perhaps in this subsection. It would also benefit the authors to add a definition of the correlation coefficients and the meaning of their interpretations.

Results: There were no analyses reported per life stage of the tick (see major comments). The authors should consider adding data per life stage and/or showing whether life stage was a significant factor in the quantity and quality of extraction DNA. Reporting of results should generally follow the same order as the methods. Suggested order:

3.1 Quantification and quality of DNA

DNA quantity/yields

DNA quality

3.2 Comparison of measurement methods (GLM results do not need to a separate subsection)

3.3 Pathogen testing

Line 202: “All samples across the different DNA extraction methods yielded amplifiable *I. ricinus* DNA, as judged by qPCR.”

Lines 202-222 and Figure 1: This information seems out of place. Could this go where you report results for DNA measurements? I understand that this gives a big overview of the results, but it seems out of order compared to the rest of the subsection. Just a thought!

Line 210: Is the range really from 280-19700 ng for DS DNA? 19700 ng seems rather large, so I’m wondering if this might be a typo.

Line 210 (and elsewhere): There is usually a comma before “respectively.”

Lines 214: This statement brings up a good question – was there generally a consensus in which samples had better yield across the different extraction methods and measurement methods? Just because 16 samples were below the limit of quantification for fluorometry, did the same 16 samples also have poor readings for the other measurement methods?

Lines 226-229: I think supplementary figure 25 would be useful to include in the main text. I was wondering if there was a main figure for these data since it would be useful to visualize this particular result. I was glad to see it included in the supplementary materials, but I think it could qualify as a figure in the main text.

Lines 227 (and elsewhere): For any statements that mention a significant difference (or even not a significant difference), please include proper statistical reporting.

Figure 2: This figure is difficult to interpret, even with the current caption. What tick life stage(s) is represented in this figure? Are the columns with the same initials replicates of the same tick? Different ticks? If there are different ticks represented, notations to differentiate the different samples and/or life stages would be helpful. Alternatively, I suggest replacing this figure with a different figure that displays the different shapes described in the text (lines 239-252), and the figure caption can include similar descriptions. As is, the current figure doesn’t add much to the manuscript.

Line 249: “… (2/100). In summary, high molecular weight…”

Line 250: Where did “76%” come from and how was this calculated? I was able to calculate 73% for ammonia extracts from the text, but I’m confused how the percentages for the Qiagen kits were computed. Why was the “peculiar” sample not included in the total counts of samples that had detectable DNA? If it was not included in that total count, this should be said in the text with a reason for excluding it in the count.

Table 4: Please provide more information about the table in the caption, namely definitions for each of the columns.

Lines 256-259: This is one sentence for a whole paragraph and it is quite long. Please consider making these into two (or more) sentences to highlight your main points. In addition, in line 259 (and to an extent, line 258), what do you mean by “difference?” Difference in what? Further, what do you mean by “not confirmed?”

Lines 263-265: I think having a figure showing this would be more impactful. See major comment about making a multi-panel figure showing yields from different extraction kits and measurement methods.

Lines 269-274 (Table 5): Can you mention in the main text (probably the methods, but could also fit in the results) and the table caption how the median ratios were calculated (i.e., what measurements did you use in the ratios?)? Can you also clarify what you mean by “questionable applicability?” Why are some measurements considered to have questionable applicability and how was this decided?

Lines 279-290 (Table 6): As mentioned in a previous comment, the methods on calculating the correlation coefficients were not provided in the methods; please add this information to the methods. Some information is provided in the table caption (line 289), but this information is not complete and it should also be in the main text. Furthermore, when explained in the methods, also briefly explain how it is interpreted (i.e., what do the correlation coefficients tell us?) Line 288, what do you mean by the word “appropriate” in the sentence “measurements are considered appropriate for the material?” Finally, please remove the “.” that comes after the table in line 290.

Lines 291-294 (GLM model): What was the final (aka “best”) model that was used for interpretation or was only one model used and interpreted for this study? If other covariates were included in the model, please provide those estimates as well. I think it’s somewhat difficult to interpret the results for the model without more context in the methods (see above comment).

Line 296: “*Neoehrlichia mikurensis* and *B. burgdorferi*…” In general, the full species names at the beginning of a sentence should be written out even if it’s already been previously mentioned.

Lines 296-301 (Tables 7 and 8): The order of tables 7 and 8 should be switched; tables are ordered based on when they’re first mentioned in the manuscript text. In line 298, what statistical method was used? Please add the statistical methods to make these comparisons to the methods section and add the proper statistical reporting in the results. Now that I read it again, is this statement/comparison necessary? If you use different extraction methods to test different ticks, differences seen in the number of positive samples wouldn’t be because of the extraction methods. If you extracted one tick using the four different extraction methods, then that comparison would be made. So in this case, maybe remove the sentence altogether. In the captions for tables 7 and 8, please add how the samples were tested (qPCR?) and break down the numbers of positive ticks by life stage. Maybe something like “# nymphs/#adults positive (# total positive).”

Discussion

Line 338: What does “ca” mean in this context?

Line 339: Which extraction procedure are you referencing here? Ammonia extraction for intact ticks? Homogenized ticks? All methods? Please clarify. Similarly, please clarify which extraction method(s) you are referring to in lines 340-341.

Lines 348-350: Can you provide a reference for the scientific basis behind the hypothesis that pH could affect the viability/detection of DNA in ammonia-based extracts? In addition, why were these data not included in the methods/results? This is pretty key information for the viability of ammonia-based extracts, so I’m wondering if this information should be provided. Was this waiting period applied to all samples using ammonia-based extractions?

Line 353: While the results show that homogenizing the ticks did not result in greater DNA yield, I wonder if results would have been different had the ticks been bisected with a blade instead of being homogenized with a bead beater. Many labs bisect ticks rather than homogenizing since the latter can result in sheared DNA. Can you please address this in the discussion?

Lines 355 and 415: Instead of “underline,” I think “highlight” or “underscore” would be better utilized here.

Line 362: “… to use qPCR as our gold-standard…”

Line 366: Please define the relationship between the DNA yield measurements across the different extraction methods as mentioned in “except in the case of the Qiagen Blood and Tissue kit extracts.” Do you mean that the measurements were consistent for these extracts? Reversed compared to the other extraction methods?

Lines 366-368: The sentence needs to be rewritten. The statement written after the comma in line 368 does not make sense in its current context in the sentence. I suggest breaking up this sentence into multiple sentences for clarity.

Line 381: “… adequate for this purpose.”

Lines 386-390: Any reference(s) for these statements?

Line 392: In addition to defining “sub-optimal” conditions, please expand on the role of ethanol as a preservative. Ethanol evaporation was alluded to be a possible factor in sub-optimal conditions, but I would have liked to read more information on the role of ethanol as a preservative and whether the authors think that ticks kept in ethanol would have preserved DNA and thus led to higher DNA yields during extraction.

Line 401: “… to the site in southeastern Norway…”

Lines 401-408: As mentioned previously, reporting the prevalence of *N. mikurensis* and *B. burgdorferi* seems very out of place compared to the rest of the manuscript. There is no further explanation on why this was done nor was any information provided on why it’s important to test for these pathogens. This should be provided in the introduction and discussion. Or remove from related information on this topic from the abstract, methods, results, and discussion.

Line 413: Using the word “entirely” is pretty strong language and was not necessarily tested, please remove.

Line 420: “… which involves minimal sample manipulation.”
