# Supplementary material for: Making the best of a bad sample: Comparison of DNA extraction and quantification methods using sub-optimally stored Ixodes ricinus ticks
Source: PLoS One. 2025 May 29;20(5):e0323251. doi: 10.1371/journal.pone.0323251 (PMC12121741; doi:10.1371/journal.pone.0323251)
Supplement: Attachment — Submitted filename: Answer_to_reviewers.docx [file pone.0323251.s027.docx]

**Answer to reviewers**

The authors are very grateful to both reviewers for their comments and suggestions, their work has proven extremely valuable for the quality of the manuscript.

Journal requirements of style and file formatting has been applied to figures and the manuscript. Ethical statement has been included in the methods section and the role of the founders was clarified in the cover letter. The uncropped pictures of electrophoresis agarose gels are being supplied as supplementary material as well as the data set use for the analysis.

Answers to both reviewers’ comments are provided below immediately after each comment in italics.

**Reviewer 1**

- Introduction, line 45-46: What about these conditions (Storage in ethanol) increase DNA quality? Is the quality comparable to some of the storage solutions available on the market? – *We are not aware of any use of other preservatives for ticks.*

- Introduction, line 60: Remove the “.” After the citation. – *“.” Was deleted.*

- Methods, lines 121-131: Is the method of extraction using ammonia the standard? – *There is to the best of our knowledge no standard method for extracting DNA from ticks. We have place the QIAGEN methods first, in response another request, indicating that they are the more frequently used methods.*

- Methods, line 133 and 134-136: Why were these exceptions made? – *First exception, in the previous paragraph, the fluid was ammonia; second exception, given the nature of the hard tick’s cuticle, the lysis is normally extended to allow the release of nucleic acids.*

- Methods, line 175: Missing “.” at the end of the sentence. - *“.” Was included.*

- Methods, line 186: De-underline the “.”. - *“.” De-underlined.*

- Methods, line 190: Abbreviate Neoehrlichia following the first mention in the paper. – *Checked and modify along the document.*

- Results, line 296: Spell out the scientific name if it is the first word of the sentence. -  *Checked and corrected.*

- Discussion, line 320: Abbreviate Borrelia and Neoehrlichia following the first mention in the paper, as long as it is not the first word of the sentence. – *Checked and modify along the document.*

- Discussion, line 325: Please describe more in detail, if known, about why the Qiagen Blood and Tissue kit yielded the purest DNA. *After consulting with QIAGEN technical support, it transpires that there is no difference between the two QIAGEN kits used except for the temperature of a 10 minutes post-lysis incubation in ‘Buffer AL’ (56°C vs 70°C). This makes the difference in purity difficult to account for, and we have modified the text accordingly.*

- Discussion: line 349-350: Why is this data not included in the study? Is it included in a separate manuscript? If so, please cite it. If not, is it possible to include it in this study? *We have added a description of this experiment. See section 2.2 in the methods and section 3.1 in the results.*

- Discussion, line 362: qPCR not QPCR. - *Corrected*

- Discussion, line 370: change overestimate to overestimated. – *Changed.*

- Discussion, line 401-402: Abbreviate Borrelia and Neoehrlichia following the first mention in the paper, as long as it is not the first word of the sentence. – *Done.*

**Reviewer 2**

Major comments

One major concern with the paper is that the results are not separated by tick life stage. The authors tested nymphal, female, and male ticks, all of which may have different amounts of DNA present. This could potentially affect the statistics of the study and thusly, interpretation of the results. If the authors chose to combine the life stages during the data analysis phase due to no significant difference in the amount of DNA present across the different life stages and the data were not shown, then they should mention this circumstance and provide the data either in the manuscript or as supplementary materials. Separating the results based on life stage would be more impactful as many researchers often selectively test different life stages due to resource availability, pathogen presence in certain life stages, etc.

- *No statistical differences were observed between methods when samples were separated by life stage and sex, supplementary table 1 contains an overview or the results separated by life stage and sex by method. Data will be submitted as supplementary material separated by life stage. Additionally, the whole data set has been included in the supplementary material.*

Furthermore, the methods need additional clarifying details, especially the statistical analyses. The authors reported results for which no methods were mentioned, or the methods did not provide enough detail to clearly interpret the results. For example, the authors did not add information on the ratios they calculated to compare the DNA extraction and measurement methods; the results were explained in lines 266-274 and table 5. More specifically, what values were used to calculate the ratios? In addition, their GLM also needs more context, including information on the covariates that went into the model and the outcome variable as well as the link function and distributions used. It is difficult to interpret the results mentioned in lines 194-196 and 291-294 without the context of what went into the model. The authors also conducted a correlation analysis and produced correlation coefficients to compare the DNA measurement across the DNA extraction methods (see lines 279-290, table 6), but this was not mentioned anywhere in the methods. Information on how the coefficients were calculated (what values were used?) and how the authors interpreted the results would be helpful. While some of the methods were provided in the figure caption, the information was not in the actual main manuscript text. With a lot of missing information from the methods, the results are difficult to interpret.

- *The settings and interpretation for the GLM are now clarified in the methods and results sections. Originally only one model was used, we have now done a model selection to find the more parsimonious one based on AIC, it does not change the results.*
- *Correlation coefficients, and the calculation of ratios for the Bland-Altmann plot and table 5 has been clarified.*

Furthermore, there was no information on the role of testing for *N. mikurensis* and *B. burgdorferi* in the study. While *N. mikurensis* was used to detect PCR inhibition, this was not the case for *B. burgdorferi*. It’s reasonable for the authors to include those results in the manuscript, but that should be clearly stated as a new objective in the study because testing for those two pathogens seemed out of place when that was not the main purpose of this study. If the authors choose to keep this information in the manuscript, they need to include the significance of these pathogens in the introduction and/or discussion to justify reporting the results in the manuscript.

- *The inhibition test has been left alone as one section, followed by a section specific for pathogen detection. This has been also included in the introduction as one of the aims of the study (being able to detect naturally acquired bacteria from this material).*

Finally, the figures and tables could be improved by providing more context in the captions and/or the manuscript text. Similar to what was said previously about results reporting, it would help readers if the authors included how the data in the figures/tables were calculated, where necessary. Figure 2 doesn’t really add much to the manuscript. Instead, I suggest having a figure show the different shapes described in lines 239-252. Figure 3 could be improved if the authors included all of the DNA quantification methods in one figure to compare DNA yields per DNA extraction and quantification method. That is, take supplementary figures 21-23 and make a multi-panel figure so that readers can compare the quantities directly rather than having to pull up the supplementary figures.

- *A multi-panel figure has been included as suggested. Clarifications has been made about how the data was calculated for figures, tables and statistical analysis. Figure 2 has been replaced with a figure summary of the shapes described in the text.*

**Line-by-Line Comments**

**Abstract**: I would check the submission instructions for the journal, but the abstract should probably be one large paragraph rather than separate paragraphs. *– non specified in the instruction from the journal, nevertheless, the paragraphs were unified.*

Lines 15-19: It would be helpful to briefly include how performance was evaluated for each DNA extraction method and how yield and purity were evaluated. This would provide more context overall to the rest of the abstract. – *Information was included in the first paragraph of the abstract.*

Line 16: Which life stages of ticks were tested? - *Information was included in the first paragraph of the abstract.*

Line 20 (and elsewhere): There are several instances where the authors use adverbs that may not necessarily be useful in the sentence’s context and/or cannot be quantified. In this case, “very” does not need to be included. The authors should go through and remove these adverbs from the manuscript accordingly. *– noted and corrected.*

Line 25 (and elsewhere): “… drop spectrophotometry…” *– Corrected.*

Line 26: What do you mean by “correlated poorly?” What values were compared for the correlation and how were the correlations calculated? – *Clarification was added*

Lines 28-29: Which methods were used to detect *N. mikurensis* and *B. burgdorferi*? *– Information included.*

**Introduction**

Line 60: “… hydrolysis method (11-13) has the advantage…” *– done.*

Line 60 (and elsewhere): Is it “ammonia hydroxide hydrolysis” or “ammonia hydrolysis?” There are several instances where either of the terms are used, but it would be better to stick with one term and to use it consistently throughout the manuscript. *– Changed.*

Line 71: “… for pure double-stranded (DS) or single-stranded (SS) DNA, and corrections…” *- Change has been done.*

Lines 70-78: The sentences need to be restructured as they are quite long-winded and confusing to read. I suggest something like this – “While this method is quite accurate for pure double-stranded (DS) or single-stranded (SS) DNA and corrections for the presence of moderate amounts of protein are available, A260 will not be an accurate measure of DNA concentration if the sample contains a mixture of DS and SS DNA (whose A260s differ) or if other substances containing aromatic rings, such as ATP, RNA, and phenol are present. Further, the A260:280 ratio is not a foolproof measure of molecular purity as many impurities have no absorbance in this region. It would be possible to construct a mixture of aromatic compounds having an A260:280 ratio of 1.8 while containing no DNA at all, for example.” This is also where you have the first mention of double-stranded and single-stranded DNA, so only the abbreviations can now be used throughout the rest of the manuscript. *– Suggestions applied.*

Lines 101-102: “… two related commercial DNA extraction kits that use silica membranes…” *– Suggestion applied.*

Line 104 (and 392): It would be helpful to explicitly define “sub-optimal” conditions either here in this line, methods, or both. This was alluded to earlier in the introduction (lines 44-47), but it should be more clearly defined.  *– Sub-optimal has been clarified in the introduction and discussion.*

**Methods**

Line 113: Could ticks still submerged in ethanol have more DNA preserved? Did any samples have ticks still submerged in ethanol when it was time to conduct DNA extractions or were all considered dry? If submerged ticks were used, this could have affected the results of the DNA extraction and quantities. Please provide more information, maybe an approximate percentage of ticks that were still in ethanol prior to the extractions- *~90% were dry, the still submerged or partially submerged ticks were randomly extracted with the four methods. Information has been included in the manuscript.*

Lines 121-131: Please provide citations for the extraction methods using ammonia. – C*itation provided.*

Line 139 (and elsewhere): Should “concentration” be replaced with “quantity?” In most cases, concentrations are not the same as quantities. - *We have reviewed the manuscript and replaced concentration with amount or yield where appropriate. However, instrument measurements reported concentration in ng/µl and in these cases, we do not feel it would be appropriate to substitute 'yield'.*

Lines 139-177: The order of the methods to quantify and evaluate DNA should be the same order in which they are introduced in the introduction. Similarly, the results should follow a consistent order as well, where appropriate. In addition, please provide the amounts of extracted DNA used for each DNA quantification method. – *The order of the methods used to quantify and evaluate DNA has been modified as suggested. The amount of DNA employed in each test has also been included in the text.*

Lines 140-142: Please add more information on how the gel electrophoresis was run. Include information such as the voltages used, amount of sample used per well (see previous comment) and time used to run the gels. – *Information included.*

Lines 163-165 and Tables 2 and 3: Tables should be switched, where the table of primers is listed first and then the PCR settings. In addition, a brief description about *5.8s* rRNA should be provided (what is it? What does it control?). A reference from the literature describing *5.8S* rRNA, its primers, and protocol should also be provided in the main text and not just in the table. – *References have been included in the main text and a brief description of 5.8S has been provided.*

Lines 172-175: “… as an anomalous amplification curve.” Also, please provide a reference for this sentence. – S*entence has been re-written, and a reference provided.*

Lines 178-186: This section seems very out of place compared to the rest of the manuscript. I understand the purpose of including *N. mikurensis* as a way to test for PCR inhibition, but then why include *B. burgdorferi* if that was not going to be used in the same way as *N. mikurensis*? – *Inhibition test and pathogen detection have been divided into two different sections.*

Line 183: Were all samples spiked with pNeo? Or just some? If only some were spiked, how were they selected? – *All samples were spiked, clarification included in text.*

Lines 183-186: Can you provide more information on PCR inhibition and how it relates to sample spiking in the introduction and/or briefly in the methods? - *We have added a description of the spiking method and provided references in the introduction.*

Line 186: Remove the underline from the period at the end of the sentence. – *Done.*

Lines 188-193: The way the sentence is currently written, it sounds like you are comparing the listed measurements to each other rather than comparing the measurements of the different extraction methods. If the latter is the case, please rephrase the sentence for clarity. In addition, “results are presented in Supplementary Table 1 and corresponding figures” can be deleted. This can be mentioned in the actual results section. Furthermore, supplementary table 1 can probably be moved to the main text as this is interesting information. If it’s moved to the main text, you should bold or add notations that mark significant differences. – *The sentence has been re written. Supplementary table 1 was moved to the main manuscript and it is now Table 5. It is referred in the text together with Figure 2 (multi-panel now) to show the statistical support estimated by Kruskal-Wallis and pairwise comparisons by Mann-Whitney-Wilcoxon tests for a better readability.*

Line 194: “A generalized linear model…” *– Done.*

Lines 194-196: This sentence does not make sense. More information on the GLM needs to be provided in general, see major comments. – *Information about the GLM has been provided.*

Line 198: R was used to do the statistics, but please also provide R packages that were used to calculate any measurements or produce data. In addition, information on Excel was not provided here even though a function used in Excel was mentioned in a table to calculate correlation coefficients (lines 288-289). Information on how the correlation coefficients were calculated were also not provided (what was the equation and what values were used?), but they need to be mentioned somewhere in the methods, perhaps in this subsection. It would also benefit the authors to add a definition of the correlation coefficients and the meaning of their interpretations. – *Now we have included complete description on how the coefficients were calculated in the methods section, and a short description on how they are interpreted was included in the table caption and the results section.*

**Results**: There were no analyses reported per life stage of the tick (see major comments). The authors should consider adding data per life stage and/or showing whether life stage was a significant factor in the quantity and quality of extraction DNA. Reporting of results should generally follow the same order as the methods. Suggested order:

3.1 Quantification and quality of DNA

DNA quantity/yields

DNA quality

3.2 Comparison of measurement methods (GLM results do not need to a separate subsection)

3.3 Pathogen testing

– *Differences by life stage were indeed present. Nevertheless, when comparing extraction and quantification methods, separating the data by life stages made no difference, supplementary table 1, previously Supplementary table 2 has a general overview of all results by life stage and S2 Table contains the entire data set (where the life stage of each sample is indicated). The order of the paragraphs has been modified.*

Line 202: “All samples across the different DNA extraction methods yielded amplifiable *I. ricinus* DNA, as judged by qPCR.” *– Applied.*

Lines 202-222 and Figure 1: This information seems out of place. Could this go where you report results for DNA measurements? I understand that this gives a big overview of the results, but it seems out of order compared to the rest of the subsection. Just a thought! – *The order of the paragraphs has been modified.*

Line 210: Is the range really from 280-19700 ng for DS DNA? 19700 ng seems rather large, so I’m wondering if this might be a typo. *– Not a typo.*

Line 210 (and elsewhere): There is usually a comma before “respectively.” – *Solved.*

Lines 214: This statement brings up a good question – was there generally a consensus in which samples had better yield across the different extraction methods and measurement methods? Just because 16 samples were below the limit of quantification for fluorometry, did the same 16 samples also have poor readings for the other measurement methods? - *The 16 samples as a group had poorer readings in the other measurements, but it was not the case in general for the rest of the samples. Poor and better performances were observed for the same samples in different measurement methods.*

Lines 226-229: I think supplementary figure 25 would be useful to include in the main text. I was wondering if there was a main figure for these data since it would be useful to visualize this particular result. I was glad to see it included in the supplementary materials, but I think it could qualify as a figure in the main text. – *It will be included in the figure 2 as a subfigure in a multi-panel as suggested in major comments.*

Lines 227 (and elsewhere): For any statements that mention a significant difference (or even not a significant difference), please include proper statistical reporting. – *Proper statistical reporting has been provided by reference to tables, in order to keep the text comprehensible.*

Figure 2: This figure is difficult to interpret, even with the current caption. What tick life stage(s) is represented in this figure? Are the columns with the same initials replicates of the same tick? Different ticks? If there are different ticks represented, notations to differentiate the different samples and/or life stages would be helpful. Alternatively, I suggest replacing this figure with a different figure that displays the different shapes described in the text (lines 239-252), and the figure caption can include similar descriptions. As is, the current figure doesn’t add much to the manuscript. *– A new figure has been included (Fig 4), to summarize the different patterns as suggested.*

Line 249: “… (2/100). In summary, high molecular weight…” – *Applied.*

Line 250: Where did “76%” come from and how was this calculated? I was able to calculate 73% for ammonia extracts from the text, but I’m confused how the percentages for the Qiagen kits were computed. Why was the “peculiar” sample not included in the total counts of samples that had detectable DNA? If it was not included in that total count, this should be said in the text with a reason for excluding it in the count. – *The sample is now included in the calculation*.

Table 4: Please provide more information about the table in the caption, namely definitions for each of the columns. – *More information has been included in the caption.*

Lines 256-259: This is one sentence for a whole paragraph, and it is quite long. Please consider making these into two (or more) sentences to highlight your main points. In addition, in line 259 (and to an extent, line 258), what do you mean by “difference?” Difference in what? Further, what do you mean by “not confirmed?” -*The sentence has been split, and the last two lines has been modified to clarify the meaning: Only the two Qiagen extraction methods showed a significant difference in yield, when judge by qPCR, but this difference was not observed by other measurements such as spectrophotometry or fluorometry.*

Lines 263-265: I think having a figure showing this would be more impactful. See major comment about making a multi-panel figure showing yields from different extraction kits and measurement methods. – *The multi-panel figure will be included.*

Lines 269-274 (Table 5): Can you mention in the main text (probably the methods, but could also fit in the results) and the table caption how the median ratios were calculated (i.e., what measurements did you use in the ratios?)? Can you also clarify what you mean by “questionable applicability?” Why are some measurements considered to have questionable applicability and how was this decided? - ‘*Applicability’ in this context refers to whether the method of measurement method matched the expected state of the DNA. If the measurement method was designed to detect double-stranded DNA, while the DNA was denatured (single-stranded), the method was considered inapplicable.*

Lines 279-290 (Table 6): As mentioned in a previous comment, the methods on calculating the correlation coefficients were not provided in the methods; please add this information to the methods. Some information is provided in the table caption (line 289), but this information is not complete, and it should also be in the main text. Furthermore, when explained in the methods, also briefly explain how it is interpreted (i.e., what do the correlation coefficients tell us?) Line 288, what do you mean by the word “appropriate” in the sentence “measurements are considered appropriate for the material?” Finally, please remove the “.” that comes after the table in line 290. – *Complete information has been included in the methods section, and in the figure caption. The dot has been deleted.*

Lines 291-294 (GLM model): What was the final (aka “best”) model that was used for interpretation or was only one model used and interpreted for this study? If other covariates were included in the model, please provide those estimates as well. I think it’s somewhat difficult to interpret the results for the model without more context in the methods (see above comment). – *More information has been provided about the GLM in both, methods and results sections.*

Line 296: “*Neoehrlichia mikurensis* and *B. burgdorferi*…” In general, the full species names at the beginning of a sentence should be written out even if it’s already been previously mentioned. – *Corrected.*

Lines 296-301 (Tables 7 and 8): The order of tables 7 and 8 should be switched; tables are ordered based on when they’re first mentioned in the manuscript text. In line 298, what statistical method was used? Please add the statistical methods to make these comparisons to the methods section and add the proper statistical reporting in the results. Now that I read it again, is this statement/comparison necessary? If you use different extraction methods to test different ticks, differences seen in the number of positive samples wouldn’t be because of the extraction methods. If you extracted one tick using the four different extraction methods, then that comparison would be made. So in this case, maybe remove the sentence altogether. In the captions for tables 7 and 8, please add how the samples were tested (qPCR?) and break down the numbers of positive ticks by life stage. Maybe something like “# nymphs/#adults positive (# total positive).” – *Table’s order is switched. The sentence has been removed. Positive samples by life stage are reported now in the tables.*

Discussion

Line 338: What does “ca” mean in this context? – *It means about (English for circa)*

Line 339: Which extraction procedure are you referencing here? Ammonia extraction for intact ticks? Homogenized ticks? All methods? Please clarify. Similarly, please clarify which extraction method(s) you are referring to in lines 340-341. – *It has been clarified*

Lines 348-350: Can you provide a reference for the scientific basis behind the hypothesis that pH could affect the viability/detection of DNA in ammonia-based extracts? In addition, why were these data not included in the methods/results? This is pretty key information for the viability of ammonia-based extracts, so I’m wondering if this information should be provided. Was this waiting period applied to all samples using ammonia-based extractions? – *All ammonia extracted samples were store for at least a week after extraction prior any downstream testing. It is a well-recognized biochemical phenomenon that enzymes have an optimum pH, above or below which their activity declines. There may be many reasons for this, among them loss of structural integrity due to changes in the charge distribution of the polypeptide chain. There seems to be surprisingly little literature on the pH optimum of Taq polymerases, although all PCR buffers seem to be buffered to pH 8.3 – 8.5. We include an early reference that describes pH optima for Taq polymerase. We have added the results of the pH experiments (see above).*

Line 353: While the results show that homogenizing the ticks did not result in greater DNA yield, I wonder if results would have been different had the ticks been bisected with a blade instead of being homogenized with a bead beater. Many labs bisect ticks rather than homogenizing since the latter can result in sheared DNA. Can you please address this in the discussion? – *Bisection has been addressed in the discussion.*

Lines 355 and 415: Instead of “underline,” I think “highlight” or “underscore” would be better utilized here. – *Suggestion taken.*

Line 362: “… to use qPCR as our gold-standard…” - *Done.*

Line 366: Please define the relationship between the DNA yield measurements across the different extraction methods as mentioned in “except in the case of the Qiagen Blood and Tissue kit extracts.” Do you mean that the measurements were consistent for these extracts? Reversed compared to the other extraction methods? – *Clarification was included.*

Lines 366-368: The sentence needs to be rewritten. The statement written after the comma in line 368 does not make sense in its current context in the sentence. I suggest breaking up this sentence into multiple sentences for clarity. – *Sentence has been broken.*

Line 381: “… adequate for this purpose.” *– Corrected.*

Lines 386-390: Any reference(s) for these statements? – *We are not aware of any scholarly literature on the subject, but we have supported our statements with current prices for DNA extraction and PCR by the methods we used.*

Line 392: In addition to defining “sub-optimal” conditions, please expand on the role of ethanol as a preservative. Ethanol evaporation was alluded to be a possible factor in sub-optimal conditions, but I would have liked to read more information on the role of ethanol as a preservative and whether the authors think that ticks kept in ethanol would have preserved DNA and thus led to higher DNA yields during extraction. – *Information about ethanol as a preservative has been provided in the introduction. Sub-optimal has also been defined.*

Line 401: “… to the site in southeastern Norway…” – *Correction done.*

Lines 401-408: As mentioned previously, reporting the prevalence of *N. mikurensis* and *B. burgdorferi* seems very out of place compared to the rest of the manuscript. There is no further explanation on why this was done nor was any information provided on why it’s important to test for these pathogens. This should be provided in the introduction and discussion. Or remove from related information on this topic from the abstract, methods, results, and discussion. – *The importance of the inclusion of bacterial detection is now included in the mentioned sections of the document.*

Line 413: Using the word “entirely” is pretty strong language and was not necessarily tested, please remove. – *Removed.*

Line 420: “… which involves minimal sample manipulation.” – *Done.*
